# Synthesis, optical linear and non-linear characterization and metal ion sensing application of some novel thieno[2,3-b]thiophene-2,5-dicarbohydrazide Schiff base derivatives

**DOI:** 10.1038/s41598-024-83994-0

**Published:** 2025-01-10

**Authors:** Ahmed. M. M. Soliman, Mahmoud Abd El Aleem Ali Ali El-Remaily, Moumen S. Kamel, Alaa El-Araby, E. Kh. Shokr

**Affiliations:** 1 Department of Chemistry, Faculty of Science, Sohag University, Sohag, 82524 Egypt; 2 Department of Physics, Faculty of Science, Sohag University, Sohag, 82524 Egypt

**Keywords:** Metal ion sensor, Non-linear optics, Optical spectroscopy, Thieno[2,3-b]thiophene, Chemistry, Materials science, Optics and photonics

## Abstract

Synthesized 3,4-Diaminothieno[2,3-b]thiophene-2,5-dicarbohydrazide (DTT) Schiff base derivatives newly were synthesized by attaching with different aldehydes, deposited in thin film form by thermal evaporation technique, and characterized by UV–Visible-NIR spectroscopy, FT-IR, NMR, and elemental analysis. It is revealed that compound **4** has the highest absorption peak intensity at 586 nm. The allied absorption, dielectric, and dispersion parameters have been calculated and discussed. The obtained results manifested that compound **4** and DTT have lower (1.92 eV), and higher (3.47 eV) energy band gap values, respectively, as a result of the conjugation number effect. The high nonlinear refractive index n_2_ and third-order nonlinear susceptibility χ^(3)^ of these organic thin films are comparable with those of chalcogenide and oxide materials, making them promising for nonlinear optical systems. Compound **4** displays high sensitivity towards metal ion detection (ex. Cu^+2^, Ni^+2^, Fe^+3^, Mn^+2^, Pb^+2^, Co^+2^), suggesting its ability to be applied as a metal ion sensor and quantifying their concentration levels by means of a suitable calibration curve.

## Introduction

Due to their tunable band gap and electronic properties, organic semiconducting compounds have received increasingly technological interest and are widely introduced in different electronic and optoelectronic applications such as organic field effect transistors (OFETs), organic light-emitting diodes (OLEDs), sensors, and organic photovoltaic devices (OPVs)^1,2^. Besides, organic material devices characterized by a high efficiency-to-cost ratio offer economic applications in different optoelectronic technologies.

The most significant advantage of organic compounds consisting of thiophene rings is their easy modification, which directly affects their electronic properties, optical properties, and high charge transport characteristics^3,4^. Increasing conjugation causes red shifts, thus reducing the band gap energy and providing new optoelectronic properties for the optoelectronic industry.

The characterization of light absorption and the acquisition of various optical parameters such as absorption coefficient (α), band gap (Eg), extinction coefficient (k), and refractive index (n) are crucial parameters for elucidating the electronic structure^5,6^. Additionally, dispersion parameters and dielectric properties are important factors in designing and implementing optoelectronic applications.

Such organic materials manifest non-optical behavior when they are exposed to an intense beam of light, such as laser radiation. The non-proportionality of the polarization to the electric field becomes more evident. The non-linear refractive index n_2_ and 3rd order of non-linear susceptibility χ^(3)^ are highly important parameters in the intensive radiation application system, which are useful for achieving optical limit behavior and for a variety of applications^7,8^. The nonlinear n_2_ and χ^(3)^ parameters are mainly dependent on and enhanced by the increase of the polarizable bond length, density, and orientation with the induced electric field. n_2_ and χ^(3)^ are also motivated by the increase in material compactness and the donor–acceptor substitution capability of the $\pi$-conjugated system in the material^7,9^.

Furthermore, different metal ions perniciously influence human health, ecosystems, and all various living organisms^10–16^. On the other side, metals are the backbone of different technological fields. So detection, searching for them and quantification of their ion concentration represent highly demanded goals for environmental safety and industrial requirements.

Different techniques, such as atomic absorption spectroscopy (AAS), inductively-coupled plasma/atomic emission spectroscopy (ICP-AES), and inductively-coupled plasma mass spectrometry (ICP-MS) have been utilized. However, the available colorimetric and spectrophotometric approaches, which are based on measurements of UV–Visible absorbance or fluorescence of compounds, could be utilized as high-performance chemical sensors for metal ion detection^17,18^. The sensor work is based on detecting changes in the optical characteristics of sensitive molecules.

Besides, the procedures for synthesizing heterocyclic analogues using an efficient catalyst^21–27^ using glacial acetic acid provide an environmentally friendly chemical synthesis route with the advantages of simple operation, high efficiency, and a short response time^28,29^.

This work aims to synthesize some new heterocyclic derivatives, study, for the first time, their optical characteristics, and determine and compare the associated parameters. The capability of the compounds to be applied for metal ion sensing and non-linear optical systems has been experienced, too.

## Experimental

### Materials and methods

Using the Melt-Temp II melting point equipment, the melting points of the present compounds were recorded. Employing Bruker Alpha Fourier transformations for measuring IR spectra (FT-IR), DMSO-d_6_ as a solvent in ^1^H NMR and ^13^C NMR spectra recorded on a Bruker at 400 MHz, and TMS as an internal reference. Chemical shift (δ) values are expressed in parts per million (ppm). The elemental analysis was carried out using a PerkinElmer 240C Micro analyzer. Thin-layer chromatography (0.25 mm thick pre-coated silica plates) (Merck Fertigplatten Kieselgel 60 F254), and UV light were used for evaluating the reaction progress. Using thermal evaporation technology with a coating unit for depositing thin coatings (200 nm thick) onto ultrasonically cleaned microscope glass substrates under a vacuum of (7 × 10^−5^) torr manufactured using Technology Licensed from Edwards Ltd, Auto 306, 2014. Film thickness was controlled using the INFICON SQM-160 thickness monitor. The deposition rate was adapted to a round of 15 nm/min.

The absorbance, transmission and reflection and metal Ion sensing spectra were recorded at normal incidence in the wavelength range of 200–2500 nm using a computer programmed double beam spectrophotometer model Jasco-570 with reflectivity attachment model ISN-470 (Japan).

### Synthesis

#### Synthesizing 3,4-diaminothieno[2,3-b]thiophene-2,5-dicarbohydrazide(2)

3,4-Diaminothieno[2,3-b]thiophene-2,5-dicarboxylate **(1)**^30,31^ (0.314 g,0.1mol) and hydrazine hydrated (10ml) were heated under reflux for 1 h. Once the completion of the reaction was confirmed by TLC analysis, the obtained solid product was filtered off, washed with ethanol and crystalized by ethanol^32,33^.

#### 3,4-Diaminothieno[2,3-b]thiophene-2,5-dicarbohydrazide(2)

Yield 90%, yellow powder, m.p > 300°C, FT-IR(KBr,cm^−1^); 3440, 3358, 3322 (4NH_2_, 2NH), 3050 (aromatic), 1674 (2CO); ^1^H NMR (400 MHz, DMSO) δ 8.57 (s, 1H,NH), 6.98 (s, 2H,NH_2_), 4.37 (s, 2H,NH_2_); ^13^C NMR: δ 162.3, 136.1, 135.4, 129.2, 123.2; Chemical formula :C_8_H_10_N_6_O_2_S_2_ (286); Elemental Analysis :C,33.56; H,3.52; N,29.35; S,22.39 . Found: C,33.54; H,3.55; N,29.32; S,22.38.

#### General procedure of compounds 3-8

Reaction of compound **2** (1mmol) with isatin and substituted aldehydes (pipronal, 5-bromo salicylaldehyde, anisaldehyde, p-tolualdehyde, and benzaldehyde) (2 mmol) in 10 ml acetic acid glacial as a solvent and catalyst, was heated under reflux for 2h, the obtained products precipitated on hot, then were filtered off, washed with ethanol and crystalized by ethanol gave compounds **3-8** respectively .

#### 3,4-Diamino-N′2,N′5-bis(benzo[d][1,3]dioxol-5-ylmethylene)thieno[2,3-b]thiophene-2,5-dicarbohydrazide(3)

Color; Yellow, yield;60%, m.p > 340°C; FT-IR(KBr, cm^−1^); 3417, 3298, 3250 (2NH_2_, 2NH), 3162(aromatic), 2898(aliphatic), 1592, 1631 (2CO_amide_); ^1^H NMR (400 MHz, DMSO) δ: 11.09 (s, 2H,2NH), 7.98 (s, 2H_,_2CH), 7.62 (s, 4H,2NH_2_), 7.40 (s, 2H_aromatic_), 7.22 (d, 2H_aromatic_), 7.01 (d, 2H_aromatic_), 6.11 (s, 4H,2CH_2_). ^13^C NMR δ: 165.29, 151.07, 149.16, 148.45, 142.63, 129.24, 127.36, 123.47, 109.08, 106.10, 101.96, 98.02; Chemical Formula: C_24_H_18_N_6_O_6_S_2_ (550.57); Elemental Analysis: C, 52.36; H, 3.30; N, 15.26; S, 11.65 Found: C, 52.33; H, 3.32; N, 15.22; S, 11.63.

#### 3,4-Diamino-N′2,N′5-bis(2-oxoindolin-3-ylidene)thieno[2,3-b]thiophene-2,5-dicarbohydrazide(4)

Red, yield;75%, m.p = 310°C, FT-IR (KBr,cm^−1^); 3439,3414,3316(2NH_2_,4NH), 3120 (aromatic), 1719 (2CO), 1592,1610 (CO _amide_);^1^H NMR (400 MHz, DMSO) δ: 12.36 (s, 2H,2NH), 11.20 (s, 2H,2NH), 7.69 (s, 4H,2NH_2_), 7.30 (t, 4H_aromatic_), 7.07 (d, 4H_aromatic_); ^13^C NMR: δ 166.45, 165.09, 152.50, 144.05, 135.16, 132.63, 126.21, 122.14, 116.02, 110.95, 97.59;Chemical Formula: C_24_H_16_N_8_O_4_S_2_ (544.57); Elemental Analysis: C, 52.93; H, 2.96; N, 20.58; S, 11.788; Found: C, 52.91; H, 2.94; N, 20.57; S, 11.77.

#### 3,4-Diamino-N′2,N′5-bis(5-bromo-2-hydroxybenzylidene)thieno[2,3-b]thiophene-2,5-dicarbohydrazide(5)

Color: yellow, yield = 71.4%, m.p > 310 °C, FT-IR(KBr,cm^−1^); 3600 (2OH), 3440, 3422, 3308 (2NH_2_, 2NH), 3158 (aromatic), 1642,1597(2CO_amide_); ^1^H NMR (400 MHz, DMSO) δ 11.39 (s, 2H,2NH), 10.44 (s, 2H,2OH), 8.31 (s, 2H_aromatic_), 8.01 (s, 2H,2CH), 7.73 (s, 4H, 2NH_2_), 7.40 (d, 2H_aromatic_), 6.90 (d, 2H_aromatic_);^13^C NMR: δ 165.11, 155.96, 151.17, 137.72, 133.48, 133.20, 128.69, 127.49, 123.39, 118.94, 111.29, 97.37;Chemical Formula: C_22_H_16_Br_2_N_6_O_4_S_2_ (652.34) Elemental Analysis: C, 40.51; H, 2.47; Br, 24.50; N, 12.88; S, 9.83; Found: C, 40.53; H, 2.45; Br, 24.48; N, 12.86; S, 9.81.

#### 3,4-Diamino-N′2,N′5-bis(4-methoxybenzylidene)thieno[2,3-b]thiophene-2,5-dicarbohydrazide (6)

Yellow, yield; 83%, m.p = 330 °C**,** FT-IR(KBr, cm^−1^); 3340, 3300, 3222 (2NH_2_, 2NH) 2987 (aliphatic), 1634, 1599 (2CO_amide_); ^1^H NMR (400 MHz, DMSO) δ: 11.17 (s, 2H,2NH), 7.98 (s, 2H,2CH), 7.73 (s, 8H_aromatic_), 6.99 (s, 4H,2NH_2_), 3.81 (s, 6H,2CH_3_);^13^C NMR δ: 165.35, 160.94, 154.16, 151.08, 142.65, 129.20, 127.48,127.41, 114.91, 98.17, 55.78;Chemical Formula: C_24_H_22_N_6_O_4_S_2_ (522.60); Elemental Analysis: C, 55.16; H, 4.24; N, 16.08; S, 12.27; Found: C, 55.12; H, 4.22; N, 16.06; S, 12.25.

#### 3,4-Diamino-N′2,N′5-bis(4-methylbenzylidene)thieno[2,3-b]thiophene-2,5-dicarbohydrazide(7)

Yellow, yield = 83%, m.p = 320 °C, FT-IR(KBr,cm^−1^); 3442, 3421, 3315 (2NH_2_, 2NH), 3136 (aromatic), 2934(aliphatic), 1598,1637 (2CO_amide_);^1^H NMR (400 MHz, DMSO): δ 11.14 (s, 2H,2NH), 8.04 (s, 2H,2CH), 7.69 (s, 8H_aromatic_), 7.34 (s, 4H,2NH_2_), 2.39 (s, 6H,2CH_3_); ^13^C NMR: δ 166.36, 151.14, 148.77, 142.92, 139.89, 132.10, 130.44, 130.10, 127.60, 98.09, 21.51.;Chemical Formula: C_24_H_22_N_6_O_2_S_2_ (490.60); Elemental Analysis: C, 58.76; H, 4.52; N, 17.13; S, 13.07 Found: C, 58.72; H, 4.50; N, 17.11; S, 13.10.

#### 3,4-Diamino-N'2,N'5-dibenzylidenethieno[2,3-b]thiophene-2,5-dicarbohydrazide(8)

Yellow, yield = 90%, m.p > 300 °C**,** FT-IR(KBr,cm^−1^); 3410,3393,3300(2NH_2_, 2NH), 1635, 1594(2CO _amide_), 3133 (aromatic);^1^H NMR (400 MHz, DMSO): δ 11.37 (s, 2H,2NH), 8.05 (s, 2H,2CH), 7.82 (d, 4H_aromatic_), 7.74 (s, 4H,2NH_2_), 7.53 (t, 4H_aromatic_),7.45(t, 2H_aromatic_); ^13^C NMR: δ 165.42, 151.23, 150.98, 142.76, 134.84, 130.03, 129.48, 127.6, 127.3, 98.01; Chemical Formula: C_22_H_18_N_6_O_2_S_2_ (462.55); Elemental Analysis: C, 57.13; H, 3.92; N, 18.17; S, 13.86, Found: C, 57.11; H, 3.90; N, 18.15; S, 13.82 .

#### Preparation of solutions with different metal ions

Compound **4** (10 ppm concentration) was dissolved in 5 mL DMSO as solvent and metal ion salts (Nickel(II) Nitrate Ni(NO_3_)_2_,Cobalt(II) Nitrate Co(NO_3_)_2_, hydrated copper(II) sulfate CuSO_4_.5H_2_O,Lead(II) Nitrate pb (NO_3_)_2_, Ferric(III) Chloride FeCl_3_, Manganese(II) chloride MnCl_2_) (10 ppm concentration) were also dissolved in 5 ml deionized double distilled water. Prior to taking the absorption spectra, 2 ml of two solutions were combined and given a short period of time to rest at ambient temperature, then UV–Visible absorption spectra were run out at room temperature.

## Results and discussion

### Chemistry

Instead of the “classical” donor groups, electron-rich heterocycles such as thiophene, pyrrole and furan were used, that they can have a dual role as electron donor groups and as π-spacers^34–38^.

**Scheme 1:**
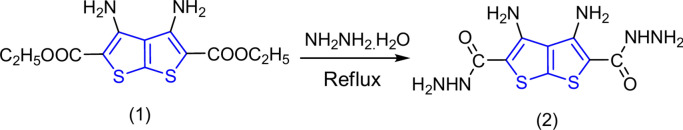
Synthesis of 3,4-Diaminothieno[2,3-b]thiophene-2,5-dicarbohydrazide.

Reaction of 3,4-Diaminothieno[2,3-b]thiophene-2,5-dicarboxylate (**1**) with hydrazine hydrate gave 3,4-Diaminothieno[2,3-b]thiophene-2,5-dicarbohydrazide(**2**) IR spectrum showed new absorption band corresponding to NH group at 3440 cm^−1^ and disappearance of ester group. Its ^1^HNMR spectrum confirms the presence of NH as a singlet at $\delta$ 8.57 ppm (Scheme 1).

**Scheme 2:**
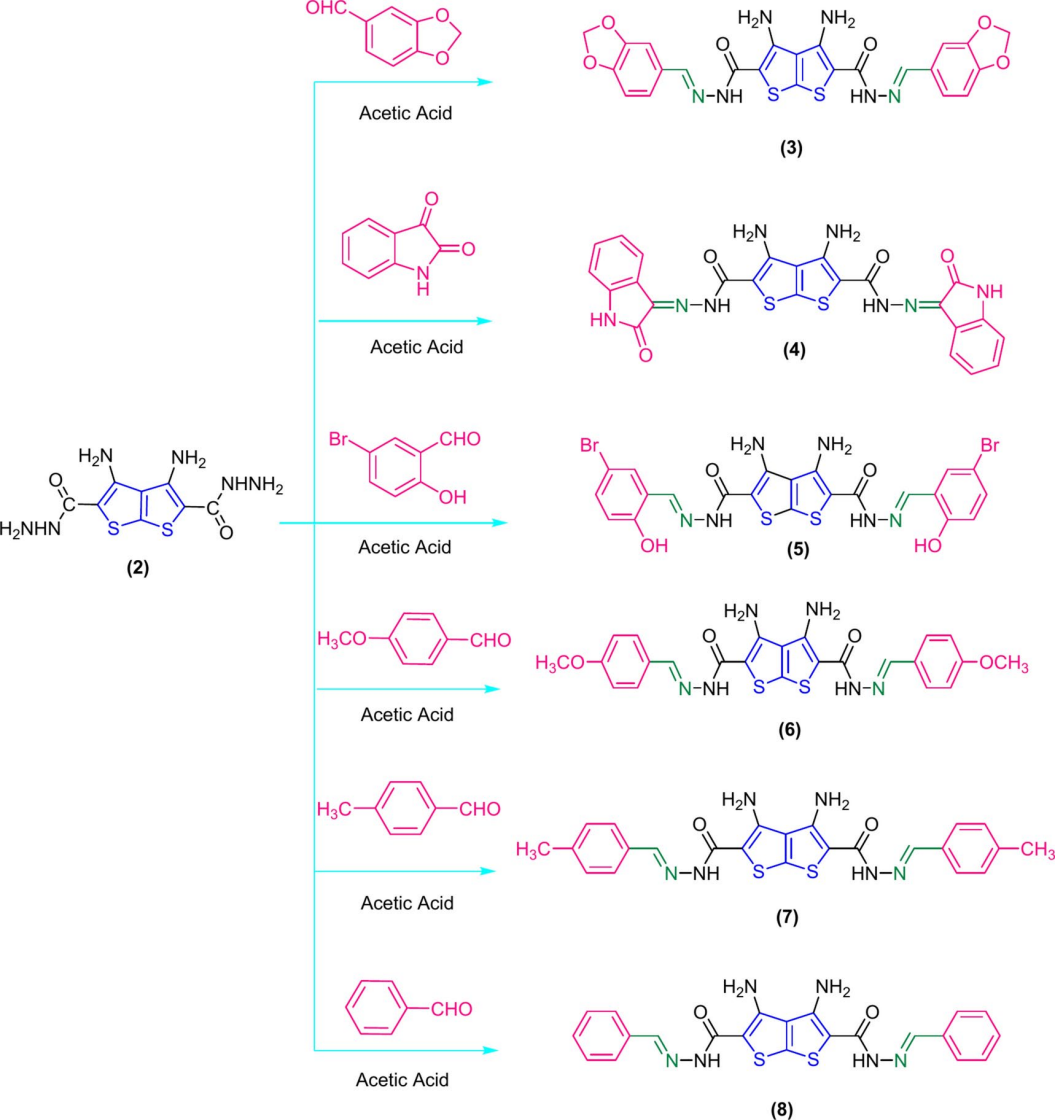
Synthesis of thieno [2,3-b]thiophene -2,5-dicarbohydrazide Schiff base derivatives **3–8**.

Thieno[2,3-b] thiophene dicarbohydrazide (DTT) was reacted with isatin and some aldehydes derivatives (pipronal, 5-bromosalicylaldehyde, anisaldehyde, p-tolualdehyde, and benzaldehyde) gave Schiff base derivatives in glacial acetic acid as a solvent and catalyst (Scheme 2). IR spectra confirmed the appearance of new absorption bands corresponding to hydroxyl substituent group at 3600 cm^−1^ for compound 5, carbonyl group at 1719 cm^−1^ for compound **4**, aliphatic substituent group at 2898 cm^−1^ for compound **3**, 2987 cm^−1^ for compound **6**, and 2934 cm^−1^ for compound **7**. ^1^HNMR spectrum of compounds **3–8** showed new signals corresponding to aromatic substituted protons signals, CH=N at $\delta$(7–8), and (7.98–8.05) ppm, respectively, aliphatic substituted protons signals CH_2_ at 6.11 ppm, OCH_3_ at 3.81 ppm, CH_3_ at 2.39 ppm for compounds **3**,**6** and **7**, respectively, and NH & OH signals at 12.36, and 10.44 ppm for compounds **4** and **5**, respectively.

## Optical characteristics investigation

### Optical spectral analysis

**Fig. 1:**
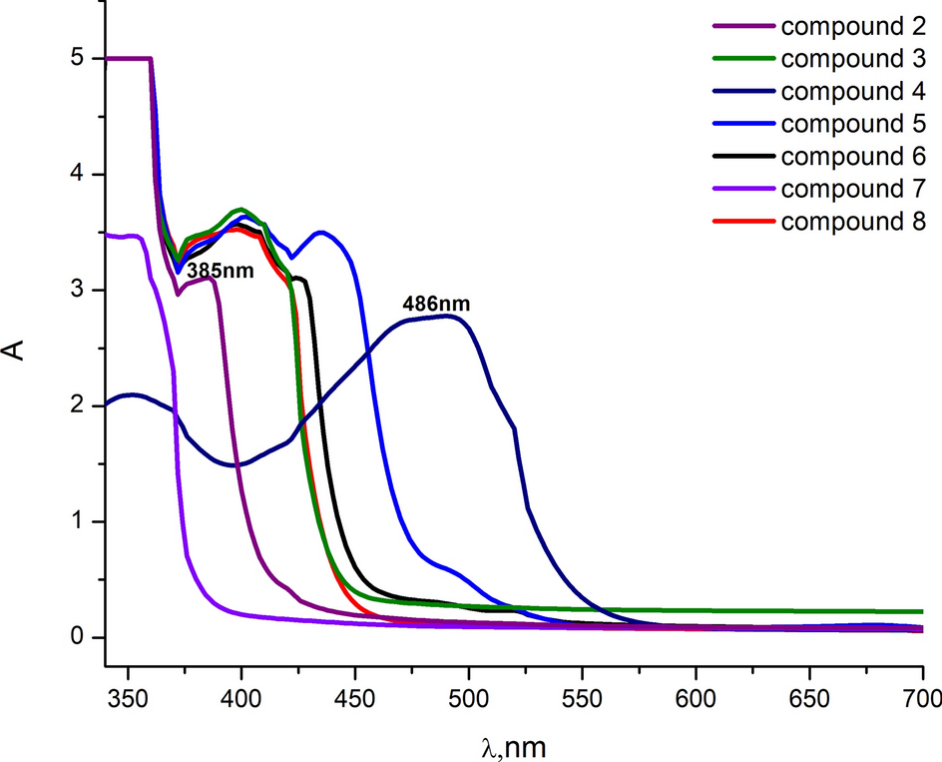
Comparison between the absorption spectra of different 3,4-Diamino thieno [2,3-b] thiophene 2,5-dicarbohydrazide derivatives dissolved in DMSO.

As shown in Fig. 1, the absorption spectra of new synthesized 3,4-Diaminothieno[2,3-b]thiophene-2,5-dicarbohydrazide (DTT) Schiff base derivatives dissolved in DMSO display major electronic absorption in the UV–Visible region**,** The observed changes in the peak intensities and positions on the spectral analysis that cause a change in the energy gap values could be dependent on the conjugation system^39–42^.

Besides, compound (**4**) has been chosen for detailed investigation since it manifests higher absorption peak intensity near maximum solar absorption wavelength (at $\sim$ 486 nm) and lower band gap energy among others associated with more light harvesting beneficial to many optoelectronic applications.

**Fig. 2:**
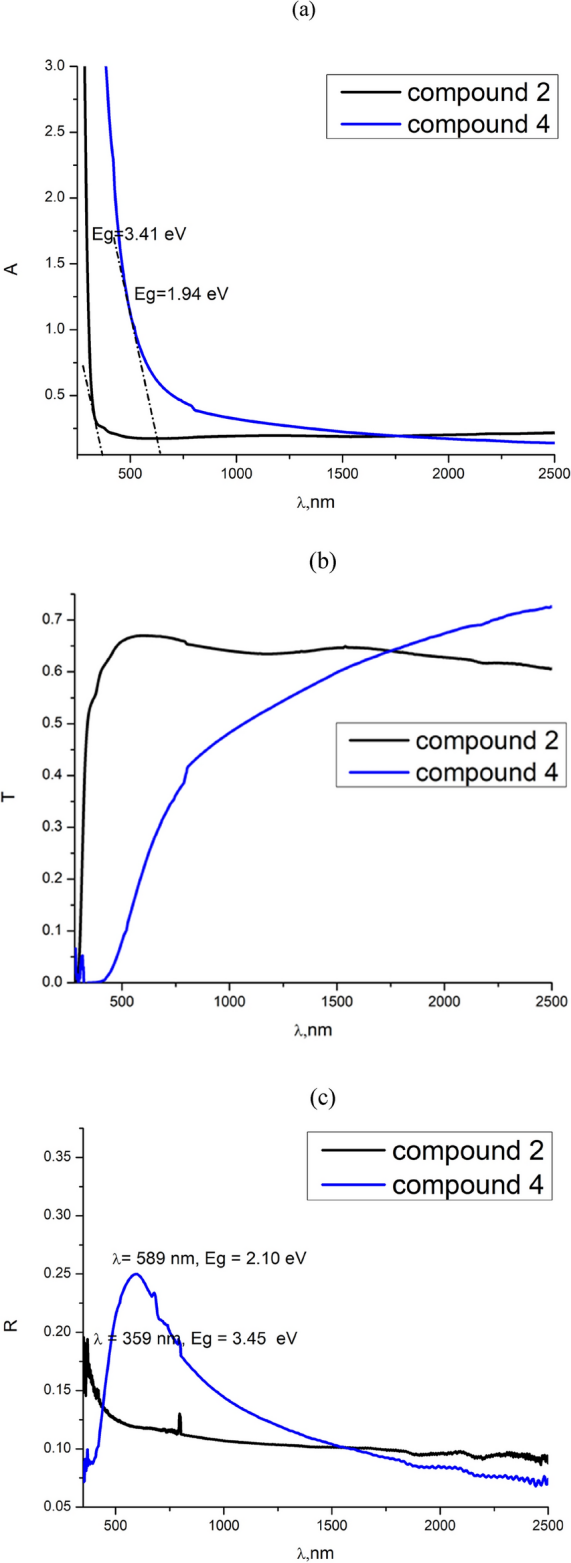
Optical absorption (**a**), transmission (**b**) and reflection (**c**) spectra of compounds **2** & **4** thin films.

The optical absorptions (A) of the start compound (**2**) and compound (**4**) thin films in the wavelength range $200\le \lambda \left(\text{nm}\right)\le 2500$ are compared as shown in Fig. 2a. To avoid the effects of glass substrates on the film transmission and reflection, the results of optical spectral measurements were considered only in the range of $\lambda >300$nm. While the optical absorption revealed by the thin film of compound **2** decreases at lower values of $\lambda$ in the UV–Visible spectral range, it monotonously decreases over the whole Vis-NIR spectral range for the film of compound **4**. The fundamental absorption edge of compound **2** lies barely in UV—region, whereas it lies in Vis—NIR region for compound **4** suggesting a decrease in band gap energy as the conjugation increases. The roughly estimated Eg values from the end absorption edges showed that Eg = 3.47, 1.92 eV for the films of compounds **2** and **4**, respectively, which can correspond to the optical transitions from HOMO to LUMO orbitals^43–45^.

As shown in Fig. 2b, the transmission spectra show relatively high T-values over the whole considered spectral range for the film of compound **2**, while T starts to increase from its very small values in the Vis—region to values as high as 75% at $\lambda >1750$ nm for compound **4**. This indicates good transparency behaviors in Vis–NIR and NIR regions for compound **2** and **4** samples, respectively, which could be emphasized by $A-\lambda$ (Fig. 2a) and $R-\lambda$ (Fig. 2c) attitudes, respectively.

These measured transmission and reflection spectra were utilized to calculate the optical parameters such as the absorption coefficient $\alpha$, the refractive index n, and the extinction coefficient k. When the multiple reflections are ignored in perfectly smooth film and substrate, the optical transmission T and consequently ${\upalpha },\text{ n and K}$ can be given by the following Equations ^52–54^;

1 $$T = (1 - R)^{2} \exp \left( { - A} \right)$$

2 $${\upalpha }=\frac{\text{Abs}}{\text{t}}=\frac{2.303}{\text{t}}\text{log}\left[\frac{{\left(1-\text{R}\right)}^{2}}{\text{T}}\right],$$

3 $$\text{n}=\left(\frac{1+\text{R}}{1-\text{R}}\right)+{[{\left(\frac{1+\text{R}}{1-\text{R}}\right)}^{2}-{(1+\text{K}}^{2})] }^\frac{1}{2}$$

and

4 $$\text{K}={\upalpha \uplambda }/4\uppi$$

where t is the film thickness.

The average values of the absorption coefficient $\alpha$, refractive index n and extinction coefficient k in the spectral UV–Visible and NIR regions and at the solar maximum wavelength $\uplambda =500$ nmwere calculated and recorded in Table 1.

**Table 1:** Values of absorption coefficient α_500_, refractive index n_500_ and extinction coefficient k_500_ measured at a solar maximum wavelength (λ = 500 nm), as well as values of the energy band gap E_g_, and Urbach energy E_u_ for TT dicarbohydrazide derivatives compounds compared with other reported results .

	${\alpha }_{500 } (nm) x{10}^{5}$	Eg (ev)	${n}_{500 } ({\text {nm}})$	${k}_{500 } ({\text {nm}})$	E_u_ (ev)	References
Compound 2	0.9	3.47	2.1	0.036	0.85	Present work
Compound 4	5.6	1.92	2.70	0.22	1.45	
Pyrrolo[2,3-b]pyrrole	2.39	2.94	1.85	0.20	–	^55^
Thieno[2,3-b]thiopheneamino ester	6.4	2.95	1.79	0.25	0.49	^30^
2-aminoanthracene-9,10-dione	–	–	1.80	0.10	–	^50^
2-amino-N-cyclohexyl-5-oxo-5H-chromeno[2,3-b]pyridine-3-carboxamide	–	3.31	2.51	0.05	0.311	^56^
N, N′-dimethyl-3, 4, 9, 10-perylenedicarboximide	–	–	1.6	0.45	–	^45^

**Fig. 3:**
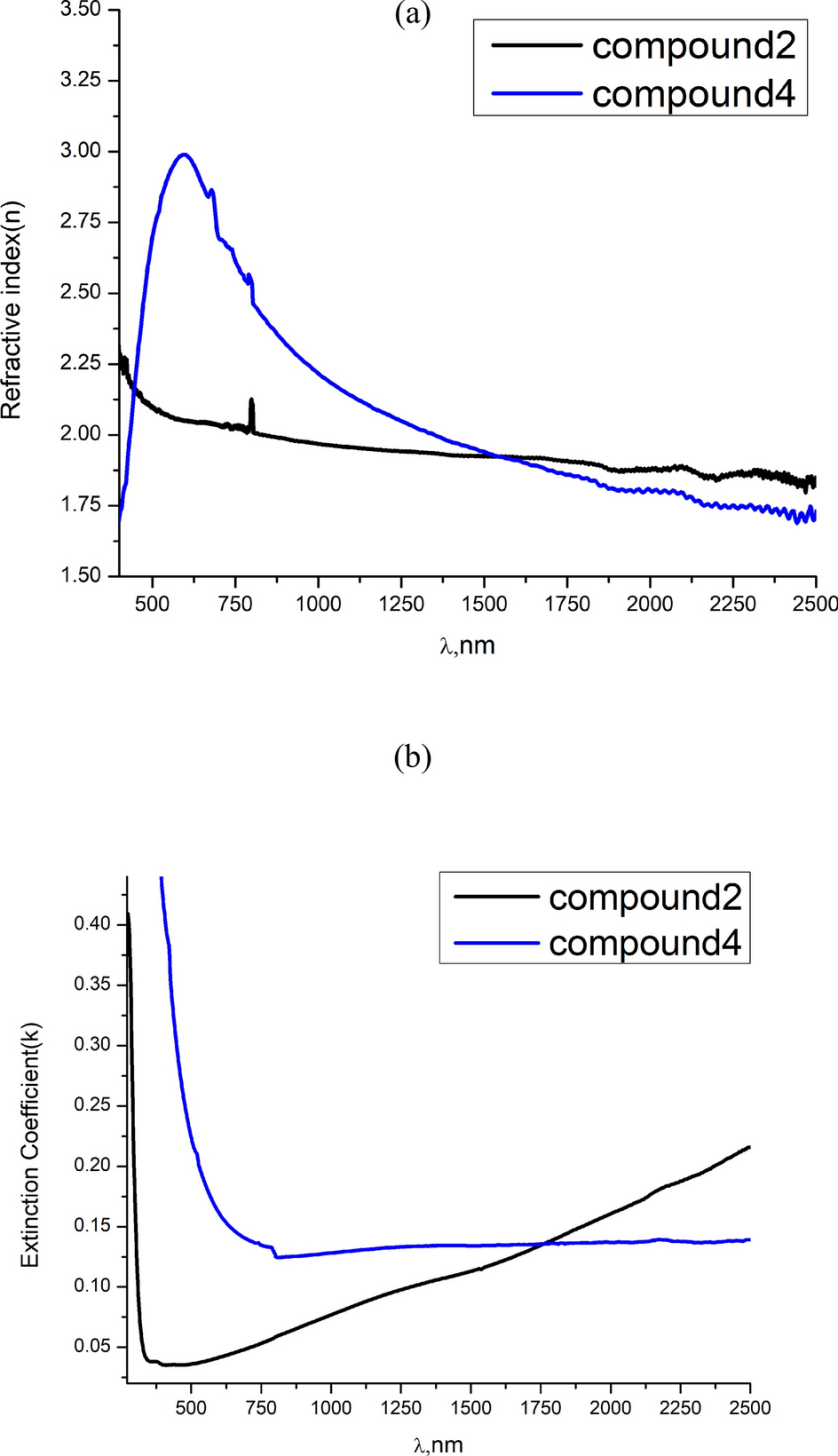
$\text{n}-\uplambda$ (**a**) and k − λ (**b**) dependence of compounds **2** and **4** thin films.

The spectral variation study of the refractive index is so important because of its crucial role in optoelectronic technology, particularly in developing optical communications and device performance^46–49^. Figure 3a depicts the spectral variation of the refractive index (n vs. $\lambda )$ of the present films. It is obvious that n manifests anomalous and normal dispersions in UV–Visible and Visible—NIR regions for compounds **2**, and **4** thin films, respectively. The energy values of 3.45 and 2.10 eV of the observed maxima for compounds **2** and **4** films are comparable to their energy band values calculated by Tauc method and recorded in Table 1. This may confirm the impression that n − $\lambda$ spectral variations can reflect the electronic fine structures of the samples^50,51^.

The extinction coefficient k, which is directly proportional to the absorption coefficient, represents the imaginary part of the complex refractive index ($\overline{n }=n+ik$). It leads to an exponential decay of the wave in the medium. As shown in Fig. 3b, k possesses high values in the region of the fundamental absorption edge ($\lambda =500$ nm) and moderate values at longer wavelengths. Such behavior of k greatly influences, particularly, the spectral variation of the dielectric and dispersion parameters.

**Fig. 4:**
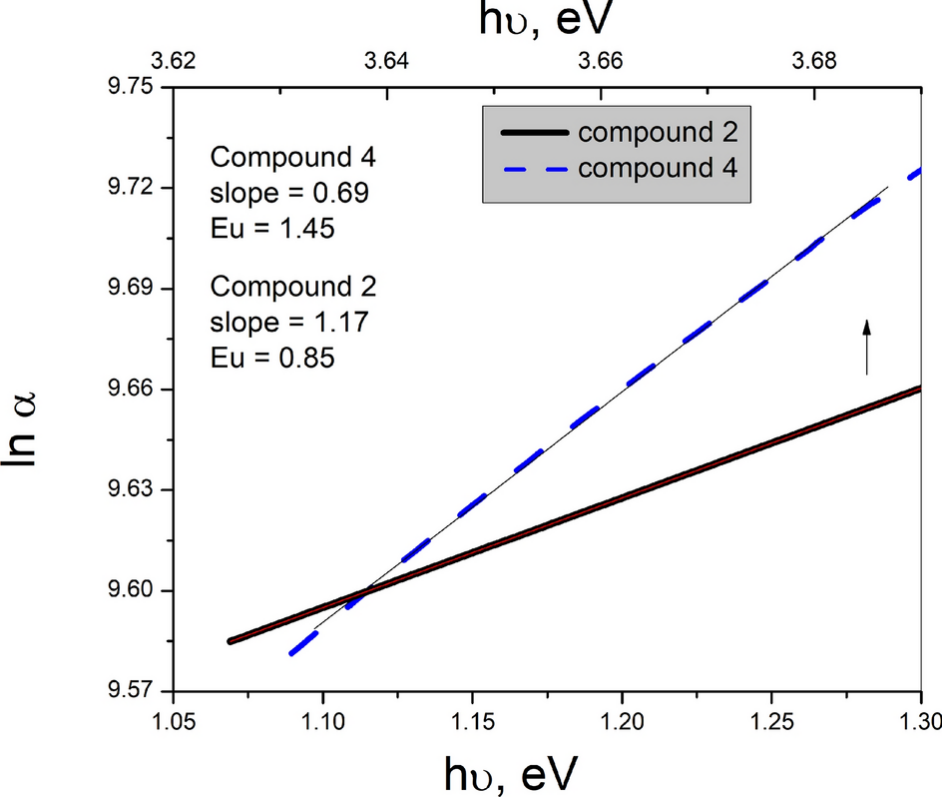
Plots lnα versus h*v* for compounds **2**, and **4** films.

In the range of $1<\alpha \left({\text{cm}}^{-1}\right)<{10}^{4},$ α varies exponentially with the photon energy (h*v*), depending on the density and tail width (E_u_) of the localized states, which are usually existed in the band gap region of the amorphous non-metallic semiconductors and verifying the following Urbach relation^57^;

$$\alpha = \alpha _{{\text{o}}} {\text{exp}}\left( {\frac{{hv}}{{E_{u} }}} \right),$$

where α_u_ is a constant and E_u_ is energy representing the measure of the tail width of the localized states. The values of E_u_ were calculated from the slopes of the lnα vs. h*v* plots (Fig. 4) and recorded in Table 1. The relatively higher values of E_u_ for the considered films compared with the reported values of other compounds (Table 1) may reflect the relatively higher glassy degree of the present films that are dependent on the microstructure and/or π-conjugation number and type of the compound film^51^.

In order to determine the probable absorption transitions and the band gap energies, one can exploit the idea of Moss criterion^52^ that the band gap value ${\text{E}}_{\text{g}}$ can be established from the value of $\uplambda$ at which the slope of the absorption coefficient at the region of absorption edge is a maximum^53,58^. The absorption coefficient for a thin film can be expressed by the following Eq. ^59,60^.

5 $$\left( {\upalpha {\text{hv}}} \right) = \upbeta ({\text{h}}v - {\text{E}}_{{\text{g}}} )^{{\text{r}}}$$

and

6 $${\text{ln}}\left( {\alpha {\text{hv}}} \right) = {\text{rln}}\left( {{\text{h}}v - {\text{E}}_{{\text{g}}} } \right) + {\text{ln}}\beta$$

where $\upbeta$ is the energy—independent constant, ${E}_{g}$ is the bandgap energy and r is an exponent that determines the type of the optical transition. By differentiation of Eq. (6) by ($\text{h}v)$, the following relation could be obtained^55^;

7 $$f\left(hv\right)=\frac{d[\mathit{ln}(\alpha hv)] }{d(hv)}=\frac{r}{hv-{E}_{g}}$$

The last equation illustrates that a maximum value of $f\left(hv\right)$ can be achieved when $\text{h}v={\text{E}}_{\text{g}}$, this concept can be utilized to determine the energy gap value and the type of its corresponding electronic transition^61^.

**Fig. 5:**
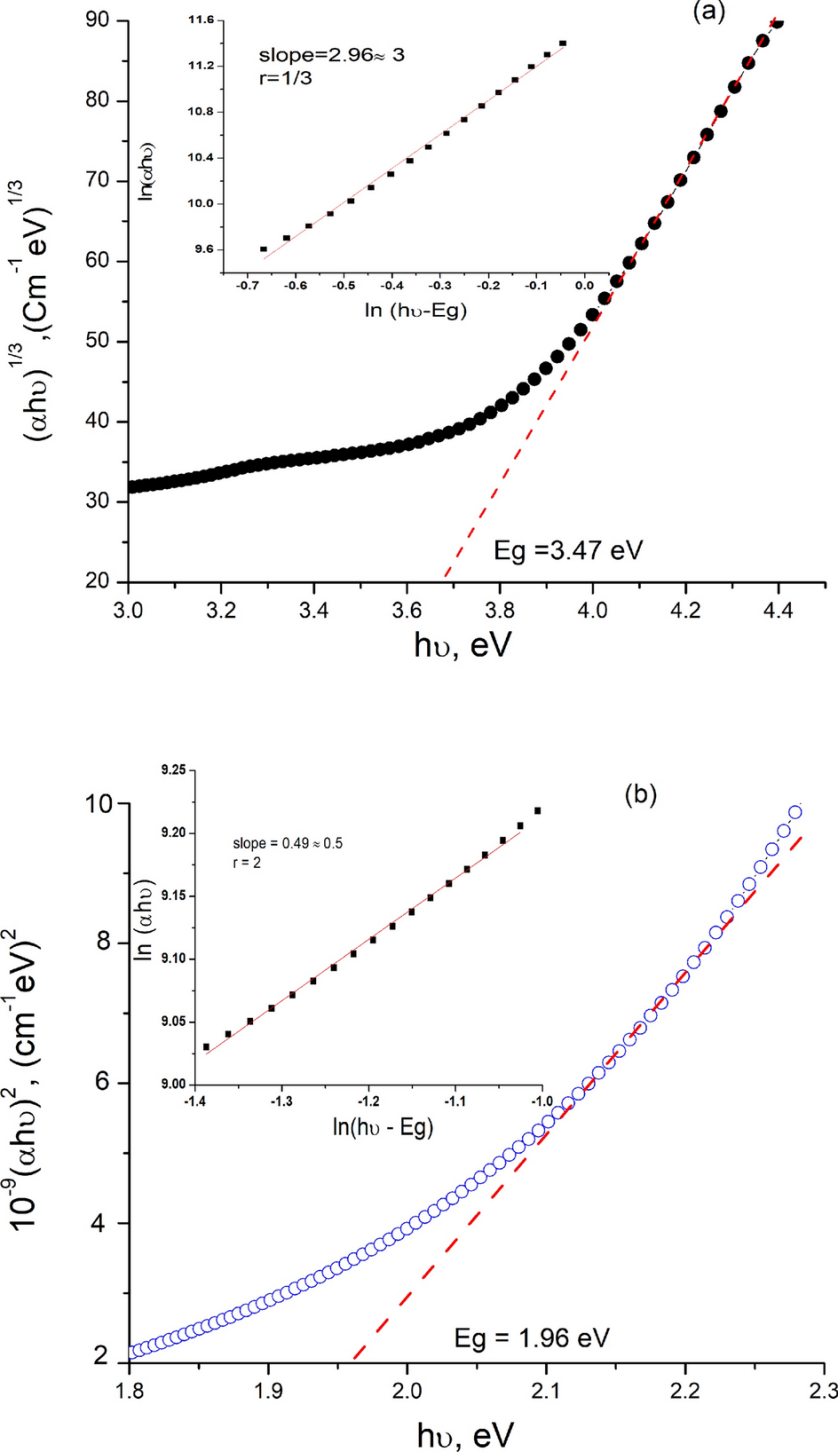
${(\alpha hv)}^\frac{1}{r}vs.hv$ plots of thin films of compounds **2** (**a**), and **4** (**b**).

On the other hand, it is thought that the optical transition theory applied by various authors, can give precise values of the transition energy as well as accurate values of the exponent r describing the types of optical transitions^50^. To deduce these values of the exponent r and identify their transition types (direct or indirect) $\text{ln}\left({\upalpha h}v\right)$ vs. $\text{ln}\left(\text{h}v-{\text{E}}_{\text{g}}\right)$ was plotted (Fig. 5a,b). The value of the exponent r for compound **2**, and **4** was deduced from the slopes and found to be close to $3 \& 0.5$ indicating forbidden indirect, and allowed direct transitions, respectively.

Thus, using $(\upalpha {\text{h}}v)^{2} {\text{vs h}}v,\left( {\alpha {\text{hv}}} \right)^{{\frac{1}{3}}} {\text{vs h}}v$ plots (Fig. 8a,b), the energy values of the band gap (${\text{E}}_{\text{g}}$) for compounds **2**, and **4** were determined from the extrapolations to $\text{h}v=0$, and found comparable with their E_g_ values reported for some conjugated organic materials, as shown in Table 1.

### Dielectric and dispersion characterization

To investigate the optical properties of a material, it is essential to introduce the frequency dependence of n, k and/or the dielectric constant.

**Fig. 6:**
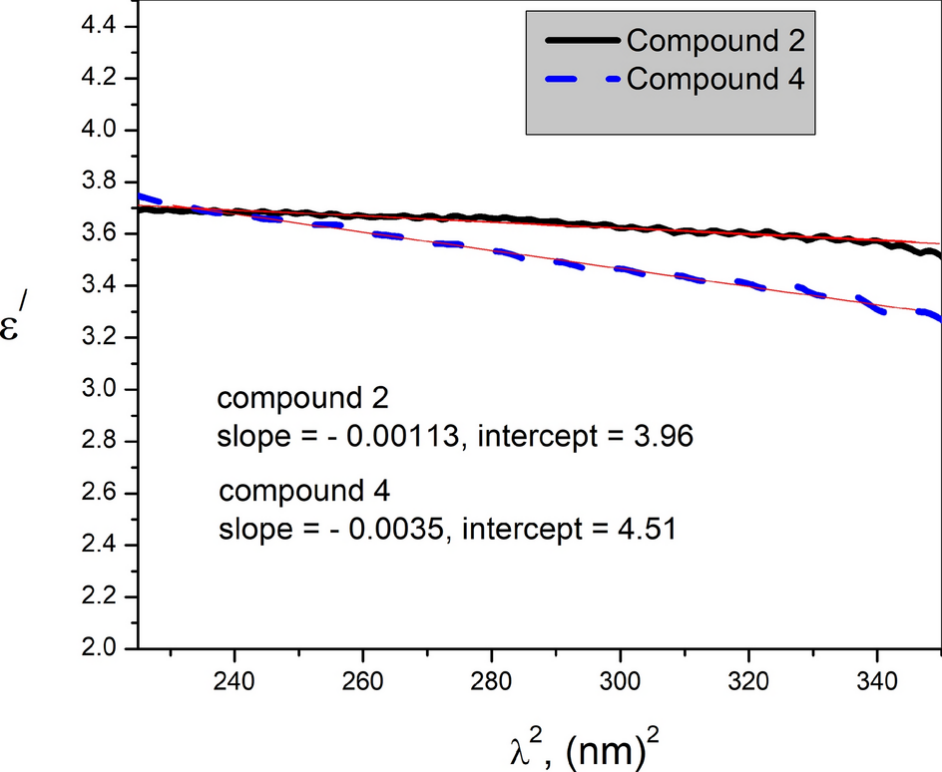
${\epsilon }^{\prime}$—${\lambda }^{2}$ plots for thin films of compounds **2**, and **4**.

In the range of normal dispersion, one can observe that the value of $\epsilon^{\prime }$ decreases with ${\lambda }^{2}$ suggesting the increase of free carriers absorption. Exploiting this behavior, some useful dielectric parameters of thin films, such as the lattice dielectric constant $\epsilon_{{\text{L}}}$, the ratio $\text{N}/\text{m}$ of carrier concentration N to m ($\text{m}$ is the ratio ${\text{m}}^{*}/{\text{m}}_{\text{o}}$ of carrier effective mass to the electron rest mass), the contribution of free carriers susceptibility ${\upchi }_{\text{c}}$ to the real dielectric constant, and the plasma frequency ${\upomega }_{\text{p}}$ can be calculated using the following relations^62^.

8 $$\epsilon^{\prime } = n^{2} - k^{2} = \epsilon_{L} - \frac{{e^{2} }}{{\pi c^{2} m_{o} }} \times \left( \frac{N}{m} \right)\lambda^{2} = \epsilon_{L} + 4\pi \chi_{c}$$

and

9 $${\omega }_{p}=\frac{2\pi C}{{\lambda }_{p}}$$

where ${\lambda }_{p}$ is the plasma wavelength, which is the $\lambda$ value at $\epsilon^{\prime } = 0$, is the electronic charge, and c is the velocity of light. All these dielectric parameters were calculated from $\epsilon^{\prime }$—${\lambda }^{2}$ plots shown in Fig. 6, and recorded in Table 2.

**Table 2:** Comparison of the dielectric and dispersion parameters of the present samples with corresponding published ones.

	E_o_ (eV)	E_d_ (eV)	${\epsilon }_{L}$	N/m* (10^19^ cm^−3^ g^−1^)	|χc|	${\omega }_{p}, {s}^{-1}$	References
Compound 2	2.51	6.13	3.97	$13.3$	0.029	3.23 $\times$ 10^14^	Present Work
Compound 4	4.51	2.70	4.51	39.0	0.034	5.25 $\times$ 10^14^	
PPY film	–	–	2.34	0.073	-	-	^65^
MV2B film	–	–	2.98	0.0032	-	-	^66^
PPY film	2.37	–	2.94	0.039	0.32	5.52 $\times$ 10^14^	^55^
TT amino ester	2.15	2.95	3.29	0.007	0.16	3.31 $\times$ 10^14^	^30^
CuTTBPc	1.88	3.42	4.14	0.0128	–	–	^67^

Furthermore, in this normal dispersion region, the dependence of the refractive index on the photon energy can be described by the following single oscillator model, introduced by Di Domenico and Wemple^63^;

10 $$\left( {{\text{n}}^{2} - 1} \right)^{{{-}1}} { } = { }\frac{{E_{o} }}{{E_{d} }} - \frac{1}{{E_{o} E_{d} }}\left( {hv} \right)^{2} ,$$

where E_o_ and E_d_ are the oscillator and dispersion energies, which are related to the overall band structure and the inter-band transition potency and structural fluctuations, respectively^64^.

**Fig. 7:**
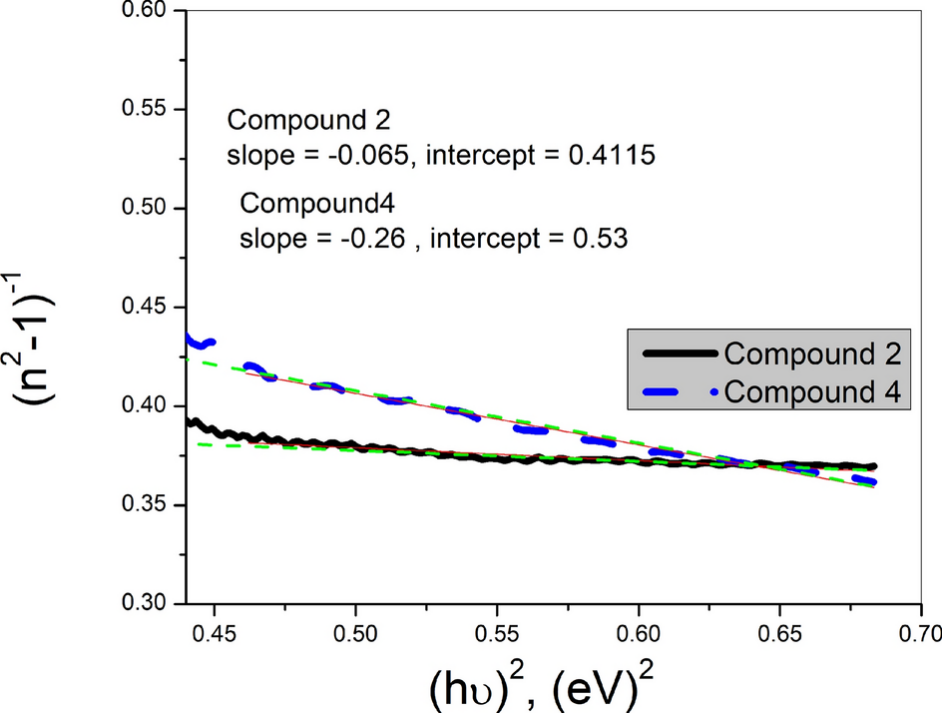
(n^2^-1)^−1^ versus (hv)^2^ plots for compounds **2**, and **4** thin films.

The (n^2^ − 1)^−1^ versus ($\text{hv}$)^2^ plots of the considered compound **2** & **4** films are shown in Fig. 7. The oscillator energy E_o_ and dispersion energy E_d_ can be determined from the slope (E_o_E_d_)^−1^ of the linear portion of the graph and the intercept (E_o_/E_d_) with the ordinate axis, where E_o_ = ${\left(\frac{intercept}{slope}\right)}^{1/2}$and E_d_ = (slope × intercept)^–1/2^.

The results of dielectric and dispersion parameters have been recorded and compared with others of some conjugated organic compounds as shown in Table 2.

### Non-linear optical characterization

The optical linear (OL) and non-linear (NLO) effects in an optical medium appear due to the harmonic and an-harmonic electron displacements around their original positions influenced by relatively low and highly intensive electric fields of optical radiation, respectively. When a material is exposed to a highly powerful electric field like that supplied by laser radiation, NLO effects become apparently dominant.

Thus, the polarization (P) which is a function of linear and non-linear susceptibilities, can be formed by the following electric field power series Equation ^68,69^;

11 $$P={\chi }^{(1)}E+{P}_{NL} ; {P}_{NL}={\chi }^{(2)}{E}^{2}+ {\chi }^{(3)}{E}^{3}+\dots$$

, where ${P}_{NL}$ is the optical non-linear polarizability, χ^(1)^ and χ^(2)^ & χ^(3)^ are the linear and second and third order nonlinear optical susceptibilities, respectively and χ^(2)^ = 0 in optically isotropic glasses.

The linear susceptibility χ^(1)^ is related to the static refractive index ${n}_{o}$, which is the value of n at h*v* = 0, by the following relation^70^;

12 $${\upchi }^{\left( 1 \right)} = { }\frac{{n_{o}^{2} - 1}}{4\pi }, n_{o} = \sqrt {\frac{{E_{d} }}{{E_{o} }} + 1}$$

The non-linear refractive index (n_2_) as well as the 3rd non-linear susceptibility ${\chi }^{(3)}$ are highly beneficial parameters for NLO—applications^71–73^. n_2_ is a crucial parameter for NLO—devices performance, while ${\chi }^{(3)}$ that describes the third harmonic generation (two photon absorption and intensity dependent refractive index), renders an important role in material utility certification for non-linear applications^74^.

χ^(3)^ is related to χ^(1)^ through the following relation^75^;

13 $$\chi^{{({3})}} = {\text{ A }}\left( {\chi^{{({1})}} } \right)^{{4}} = \frac{A}{{\left( {4\pi } \right)^{4} }}\left( {\frac{{n_{o}^{2} - 1}}{4\pi }} \right)^{4} ,$$

where A = 1.7 × 10^−10^esu^75^.

Besides, n_2_ can be deduced using the following expression^68^;

14 $${\text{n}}_{{2}} = \frac{12\pi \chi \left( 3 \right)}{{{\text{n}}_{{\text{o}}} }}$$

F-function is another important parameter that specifies the energy dispersion behavior of the non-linear absorption coefficient and identifies the energy states that are coupled^76,77^.

The degree of polarization depends on type of the bond between the two charge species and the electron density in the atomic valence shell. It is small for ionic bonds, whereas it is large for organic molecules having covalent bonds, and more electron density (the larger the atomic radius, the greater is the polarizability). Accordingly, in the considered organic compounds **2**, and **4** both weakly C–H & ${CH}_{2}$, $C({\delta }_{-}{\delta }_{-})$ & $H ({\delta }_{+}{\delta }_{+})$ and strongly $C=O$; $C({\delta }_{+}{\delta }_{+})$ & $O({\delta }_{-}{\delta }_{-})$ polarized bonds are involved, which are confirmed by FT-IR analysis. Upon polarization, the covalent bonds in an organic material can transform into more reactive polar ones resulting in polar molecules, which are consequently more reactive than regular molecules. Besides, when the molecules are oriented in the direction of the inducing electric field, their individual dipole moments participate in the net dipole moment, enlarging the polarization magnitude.

**Fig. 8:**
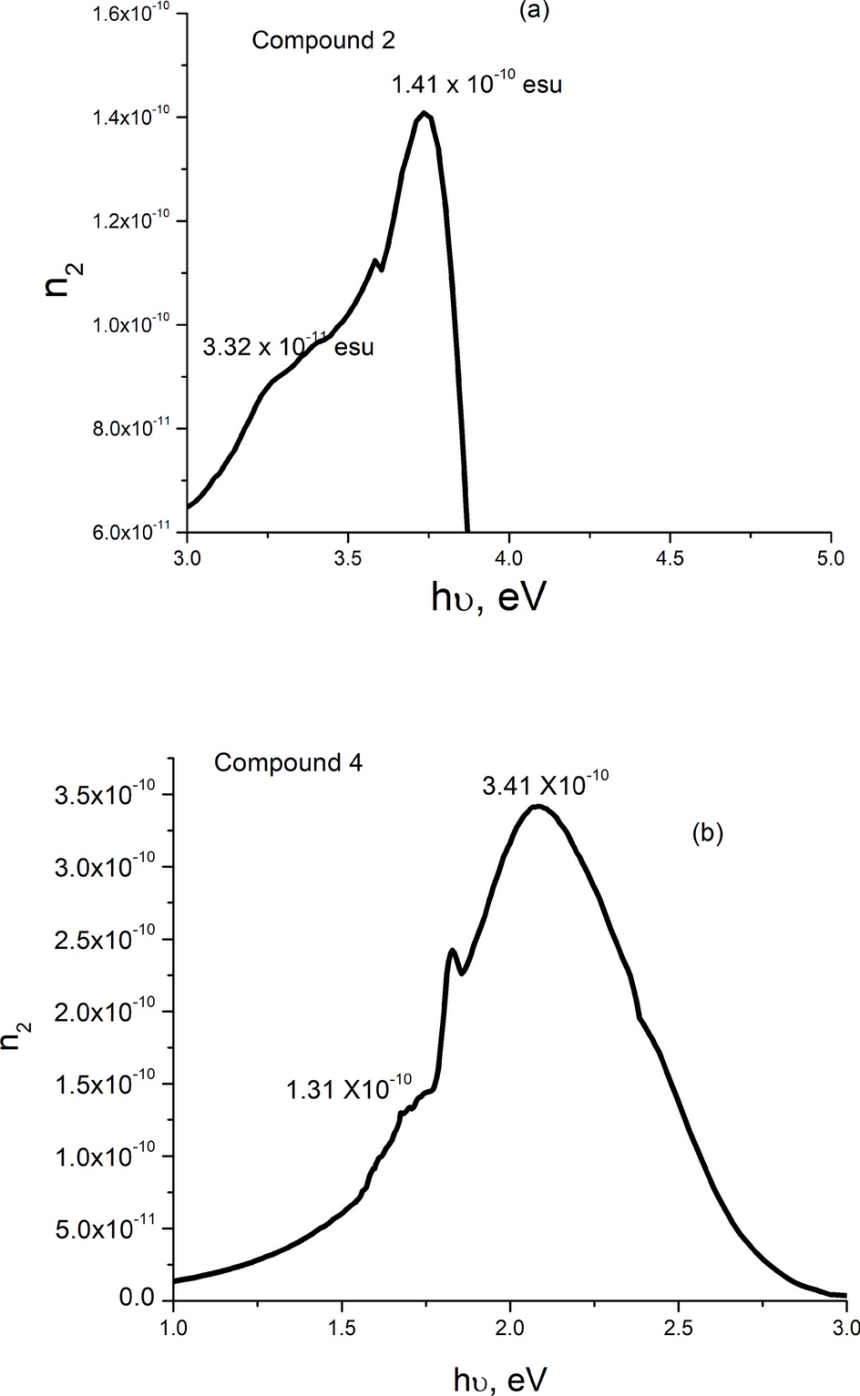
Energy dependence of non-linear refractive index n_2_ for compounds **2** (**a**), and **4** (**b**).

**Fig. 9:**
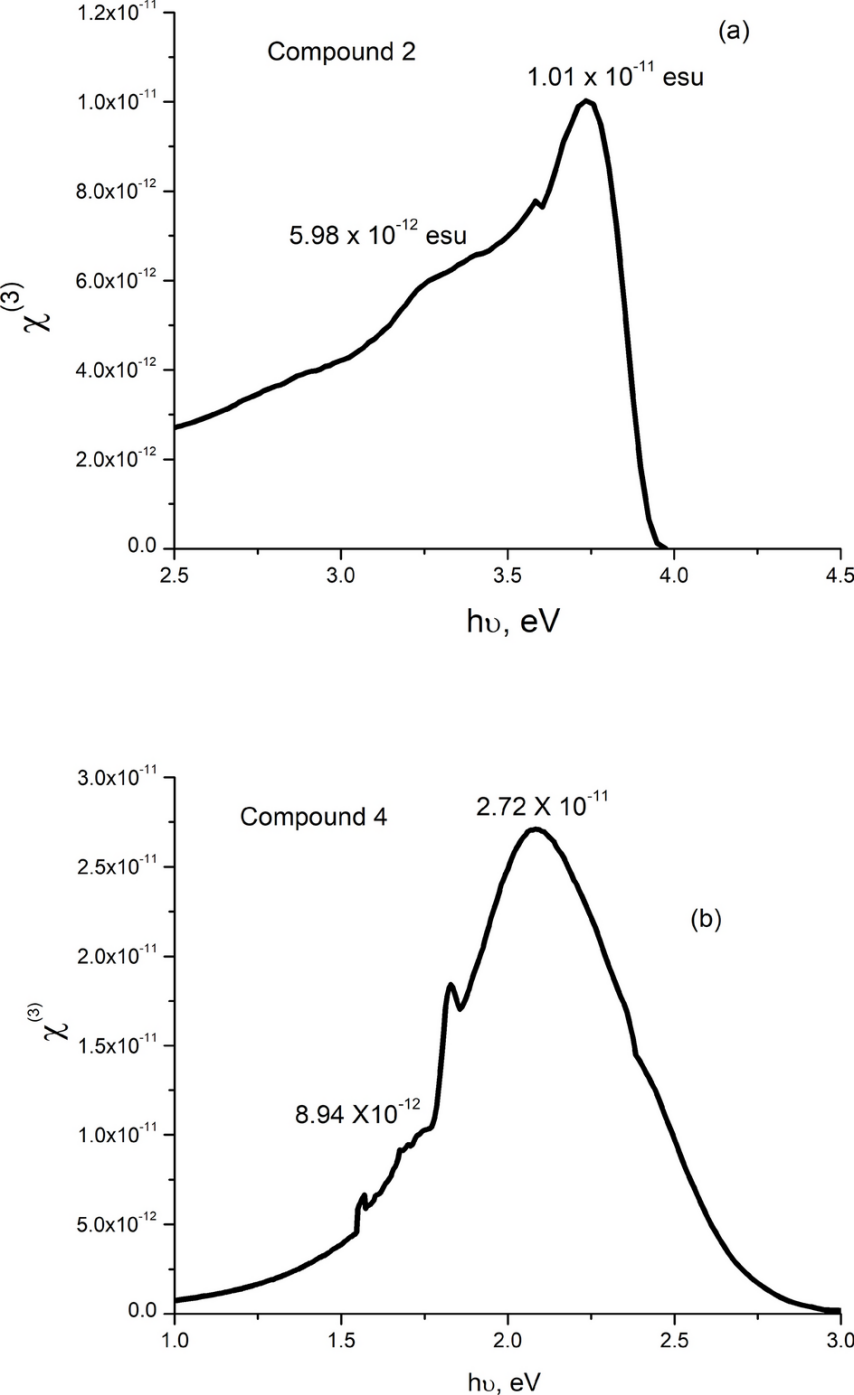
Energy dependence of third order non-linear susceptibility χ^(3)^ for compounds **2** (**a**), and **4** (**b**).

Figures 8, 9 depict the energy variations of n_2_ and χ^(3)^ for the present films of compounds 2 and 4. As shown, both energy dependences of n_2_ and χ^(3)^ have similar attitudes toward energy variation. They start with a rapid increase, passing through two coincident energy positions (weak and strong) maxima, then decrease at relatively higher energy. The relatively small peaks could be correlated to the energy activated weakly polarized C–H and ${\text{CH}}_{2}$ bonds, while the strong peaks can be attributed to the strongly polarized C=O bonds. The observed larger values of the small and high n_2_ and χ^(3)^ maxima of compound **4** than their corresponding values of compound **2** could be assigned to the relatively higher density of localized states in compound **4**^59^ (see Fig. 4 and Table 1).

**Fig. 10:**
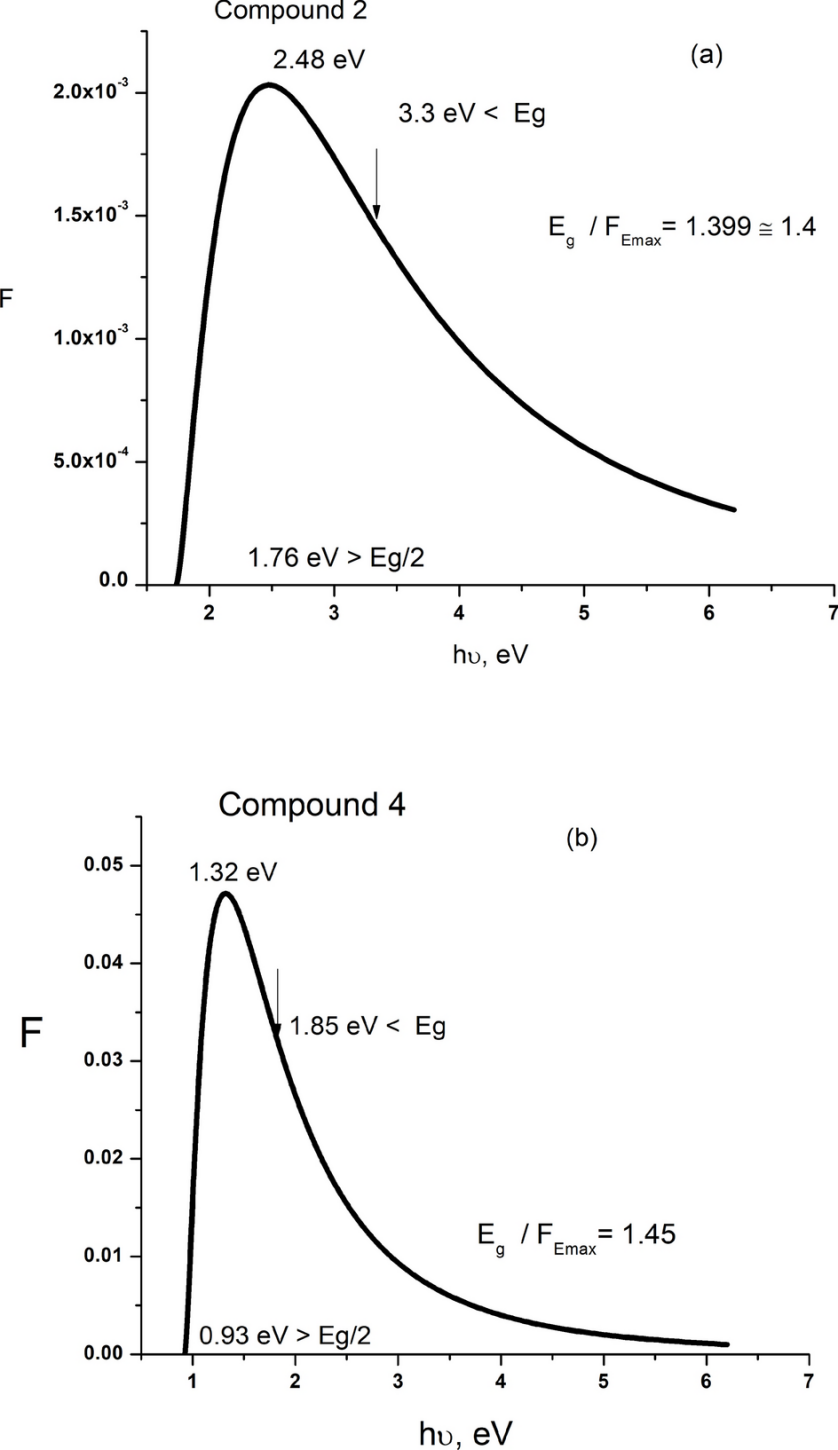
Spectral variation of F-function for compounds **2** (**a**), and **4** (**b**).

Figure 10 manifests the spectral variations of the F-function for both films of compounds 2, and 4. As shown, F starts rapidly increasing at 1.76 and 0.93 eV < E_g_/2 reaching its maximum at 2.48 and 1.32 eV and begins to decrease, inclining to constant values at 3.3 & 1.85 eV > E_g_, for compounds **2**, and **4**, respectively. Besides, the calculated values (1.4–1.45) of E_g_/F_Emax_ ratio for the present films are in its reported (1.4–1.45) range^72–76^. Such behavior of F − *hv* satisfies the condition of the third harmonic generation. The comparison of the present results of strong maximum values of n_2_ and χ^(3)^ with the corresponding published ones of some oxide, chalcogenide, and organic materials given in Table 3 indicates that the present values of both n_2_ and χ^(3)^ significantly exceed the values reported for other ones.

**Table 3:** Comparison of NLO n_2_ and χ^(3)^ maximum values of the present samples with others published for some materials.

Material	χ^(3)^ (10^−10^esu)	n_2_ (10^−10^esu)	References
Compound 2	0.27	3.41	Present work
Compound 4	0.10	1.41	
TT amino cyano + 1-naphthylamine	0.052	0.078	^51^
As_40_S_45_Se_15_	0.019	0.324	^9^
Zn depoed CdO	0.0006	0.013	^74^
CuO	0.007	0.000054	^75^
(PbS)_1−x _Zn_x_	0.016	0.25	^78^
hybrid Bixa Orellana dye doped PMMA polymer	0.00072	0.00938	^79^
gold(III) maleimide dithiolate tetraphenylphosphonium salt (Au-P)	0.000224	0.0044	^80^
gold(III) maleimide dithiolate melamine melaminium hybrid solvate (Au-Mel)	0.00047	0.0061	^80^

### Metal ion sensing application

To characterize the performance of metal sensors, such as electrochemical ones, signal reduction, selectivity coefficient, and barrier width techniques are widely used^81–83^. However, they have limitations in terms of many experimentation requirements, the unsuitability of ions with different charges, or even the difficulty of equipment existence. In the present optical measurements, the metal maximum peak of the characteristic wavelength intensity and shifting about that of pure compound 4 solutions were taken as an indication of the relative effect of the barrier width, interference, or selectivity.

Owing to the smaller band gap of compound **4** among others, it was chosen for a metal ion sensing investigation. Due to the poor solubility of compound **4** in water, its colorimetric sensing abilities were investigated in DMSO medium (10 ppm) upon the treatment with several kinds of aqueous cations, such as (Cu^+2^, Pb^+2^, Fe^+2^, Mn^+2^, Ni^+2^, Co^+2^).

**Fig. 11:**
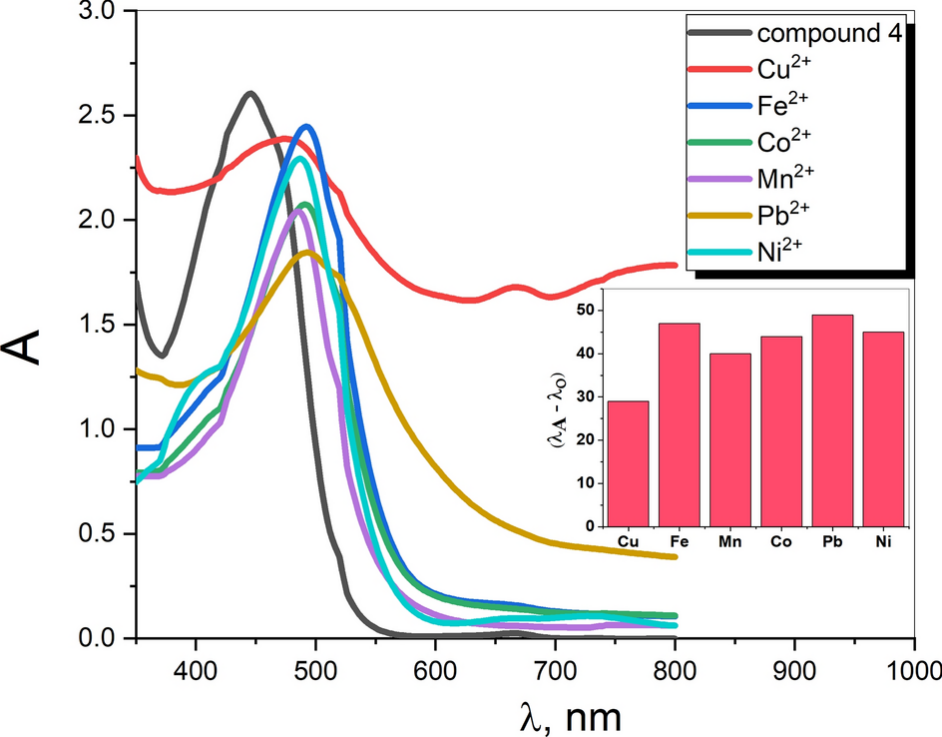
UV–Visible absorption spectra of compound **4** with some metal ions.

**Scheme 3:**
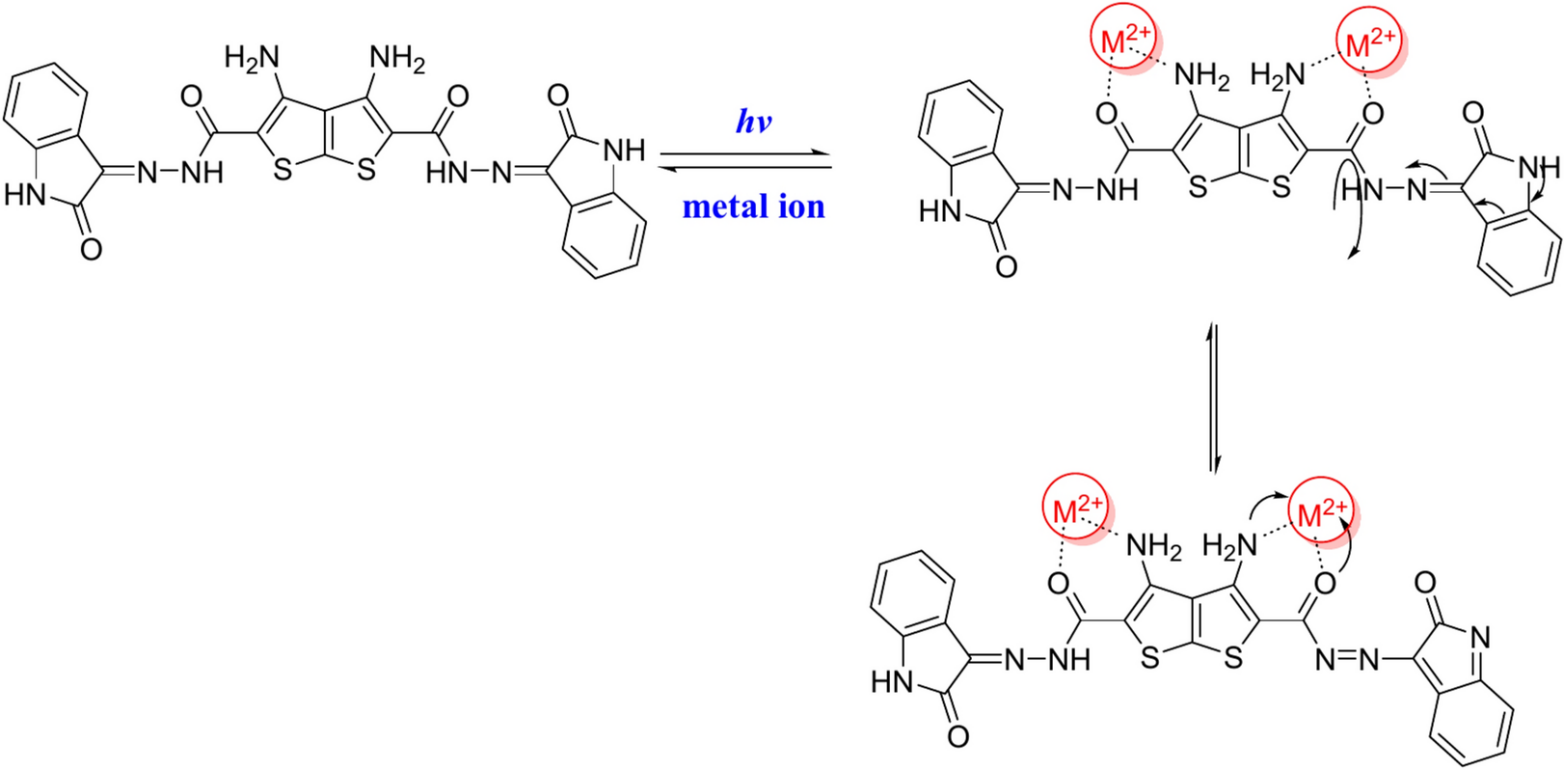
The suggested mechanism for charge transfer between compound **4** and a metal.

Figure 11 depicts the optical absorption spectra (300–800 nm) of compound **4** solution before and after the combination with the considered metal ions solutions. As shown, peaks at 445 and 492, 474, 490, 485, 489& 494 nm correspond to compound **4** solution, and compound **4** with Fe^+2^, Cu^+2^, Ni^+2^, Mn^+2^, Co^+2^& Pb^+2^ metal ion solutions, respectively. The observed red shifts in the case of existed metal ions indicate the metal detection ability of the organic compound **4**. Actually, the chromogenic reagents are composed of a donor and acceptor component, resulting in the existence of a charge transfer band in the absorption spectra. Interaction with the metal ion may have destabilized the donor component (nitrogen atom) of compound **4**, resulting in a bathochromic shift of the charge transfer band. The interaction with metal cations alters the nitrogen atom’s lone pair of electrons, demonstrating its donor properties (scheme 3). Consequently, a bathochromic shift of λ_max_ was found.

The detection sensitivity of the detector material depends on and proportional to the peak shift value, which is the difference $({\lambda }_{A}-{\lambda }_{o})$ in the peak wavelength values ${\lambda }_{A}$ and ${\lambda }_{o}$ with and without combinations of metal ions solution, respectively. As shown in (Fig. 11, inset), the present detector of compound **4** manifests the highest and lowest sensitivity for Pb^+2^ and Cu^+2^, respectively, among the considered metals ions.

#### The effect of metal ion concentration on the detector sensitivity

**Fig. 12:**
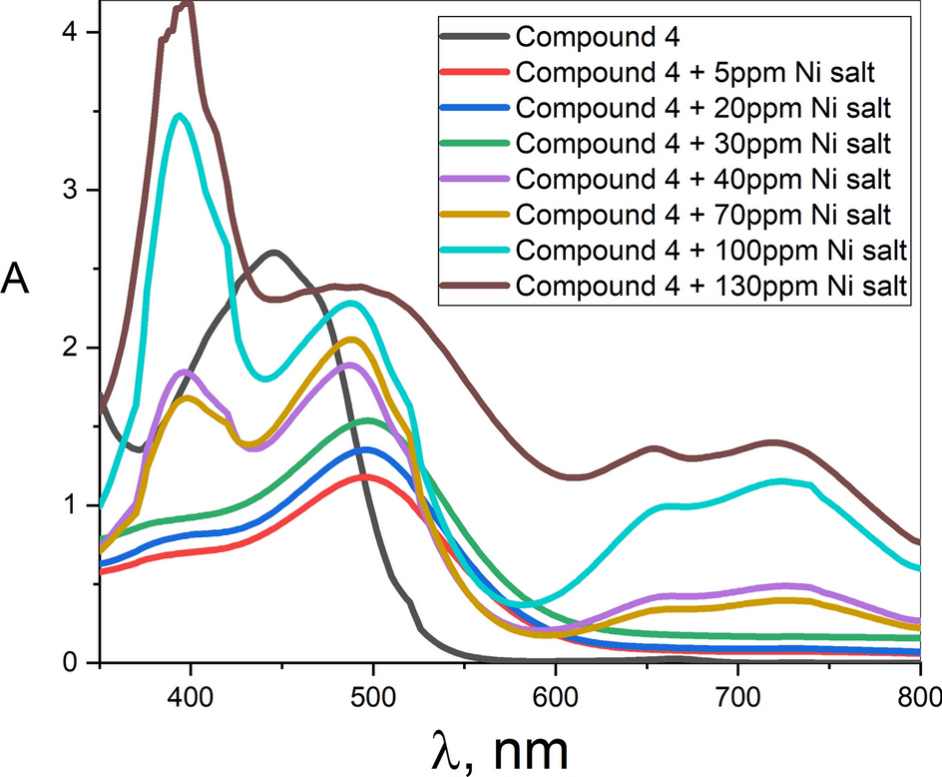
The effect of Ni^+2^ solution concentration on sensitivity of compound **4**.

In order to identify the effect of the ion concentration on the detector sensitivity, Ni-metal ion having a relatively larger peak intensity and shift was taken as an example. Figure 12 shows that, although the Ni^+2^ solution concentration increase has almost no effect on the energy position of the peak characterizing compound **4** detector, a large change in the absorption spectrum in terms of the distinct increase in the peak intensity and the appearance of additional characteristic peaks at 394, 654, and 730 nm could be observed. Such behavior shows a constant sensitivity value (${\lambda }_{Ni}-{\lambda }_{o})$ whatever the Ni-ion concentration is. On the other side, the observed increase in peak intensity with the increase of Ni-ion concentration can be employed to construct a beneficial calibration curve to quantify the unknown Ni-ion concentration in a sample.

#### The effect of pH metal ion solution on the detector sensitivity

**Fig. 13:**
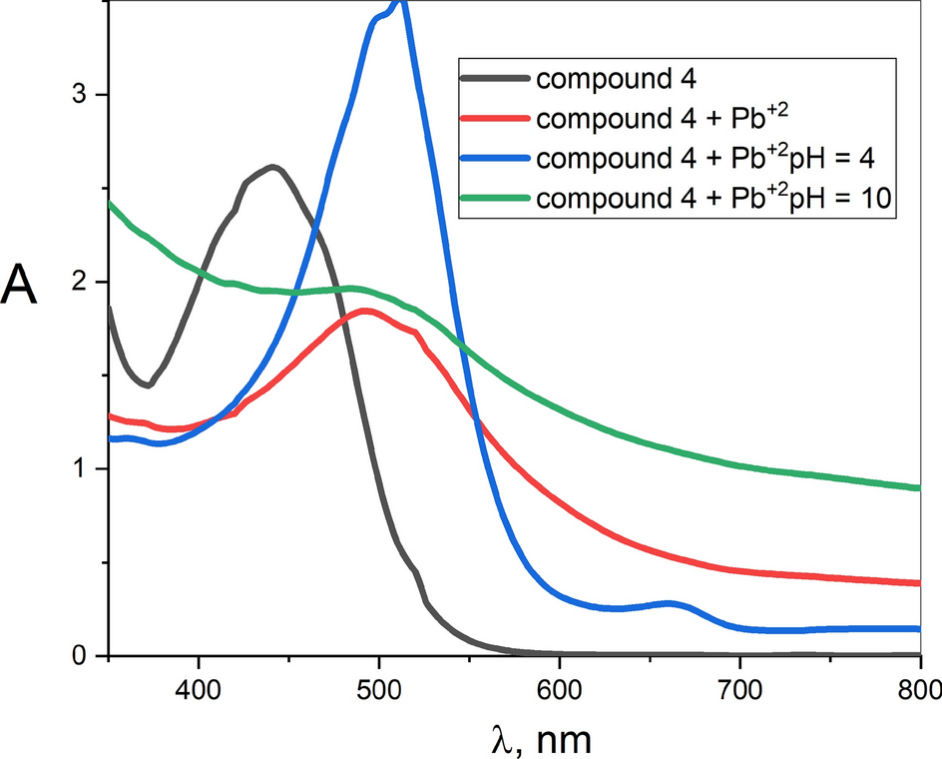
The effect of pH value on the sensitivity of the compound **4** with Pb^+2^ ion solution.

Figure 13 shows the effects of both acidic and basic solutions on the detection process of compound **4** with Pb^+2^ ion having the larger $({\lambda }_{A}-{\lambda }_{o})$ peak shift. As shown, acidic (pH = 4) and basic (pH = 10) media have no influence on the peak position but the intensity of the peak has strongly and slightly increased in acidic media and basic media, respectively. That could be because the acidic in contrast to the basic media, enhance the complex stability, indicating that the acidic media are more efficient than the basic ones for Pb^+2^ ion detection.

**Fig. 14:**
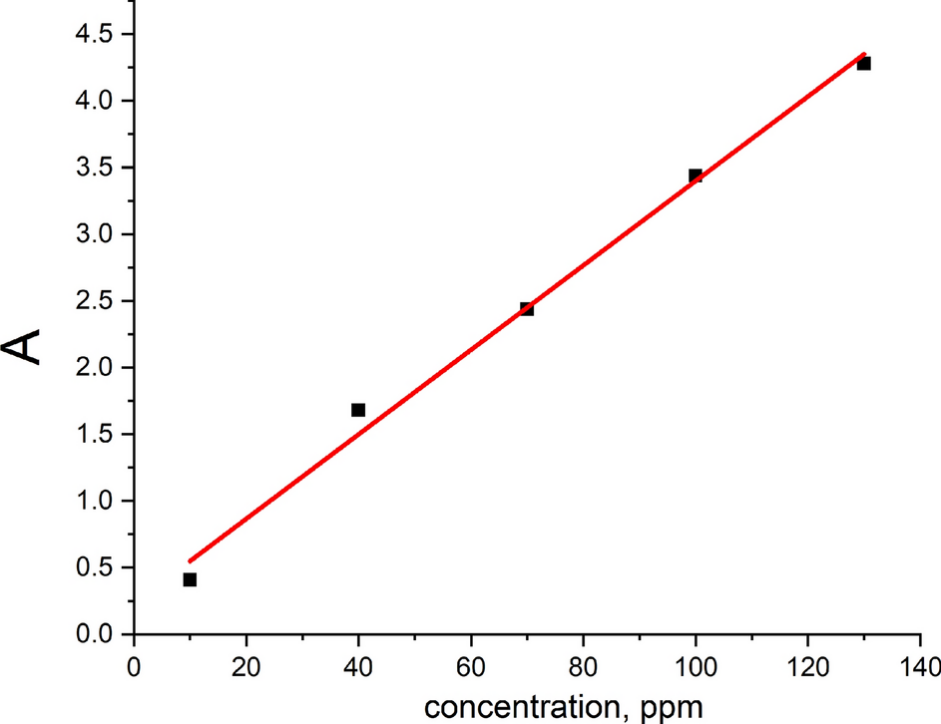
Metal ion absorption peak intensity versus Ni–ion concentration.

Since, the characteristic peak intensity is dependent on and proportional to the metal ion concentration, the plots of absorption versus metal ion concentration (Fig. 14) can be considered as a calibration curve which helps to quantify the detected metal ions.

Considering the economic criterion (device performance/cost), the calibration curve is extremely important for practical applications. It may be used to identify and measure metal ions essential for industry in any land location, as well as hazardous, poisoned, and heavy metals in water.

## Summary and conclusions

 The presently synthesized novel TT derivatives have been investigated by FT-IR, NMR, elemental analysis, and optical UV–Visible-NIR spectroscopy, which manifest major electronic absorptions in the UV–Visible region. The chosen compound **4** for more detailed investigations is distinguished by relatively high absorption peak intensity (at 586 nm). The spectral behavior of absorption, dielectric, and dispersion and their allied parameters in the films have been investigated, discussed, and compared with reported results. The non-linear parameters, such as refractive index n_2,_ and the 3rd-order non-linear susceptibility χ^(3)^ have been studied and discussed. Compounds revealed reasonably high values of n_2_ and χ^(3)^ compared with many other materials. The relatively high values of n_2_ and χ^(3)^ associated with compound **4** are larger than the values of some chalcogenide and oxide materials. The results are quite encouraging for possible applications in nonlinear optical applications such as optical switching and optical communications.

Compound **4** has manifested ability for metal ion sensing. The influence of the examination of both metal ion (Ni^+2^) concentration and pH (4 & 10) values on the Pb^+2^ detection sensitivity has revealed that it increases with the increase of Ni^+2^ ion concentration, and the detection process of metal Pb^+2^ ions in acidic media is more efficient than in the basic ones. The absorption peak strength is shown to vary linearly with the concentration of the metal ion solution under consideration. It may be used to identify and measure metal ions needed for industry on any terrestrial surface, as well as dangerous, toxic, and heavy metals in water.

## Supplementary Information

Supplementary Information.

## Data Availability

The authors declare that the data supporting the findings of this study are available within the paper and its Supplementary Information files.
