# Supplementary material for: Synthesis, optical linear and non-linear characterization and metal ion sensing application of some novel thieno[2,3-b]thiophene-2,5-dicarbohydrazide Schiff base derivatives
Source: Sci Rep. 2025 Jan 10;15:1611. doi: 10.1038/s41598-024-83994-0 (PMC11724020; doi:10.1038/s41598-024-83994-0)

^{1}H-NMR and ^{13}C-NMR of compound **(3)**

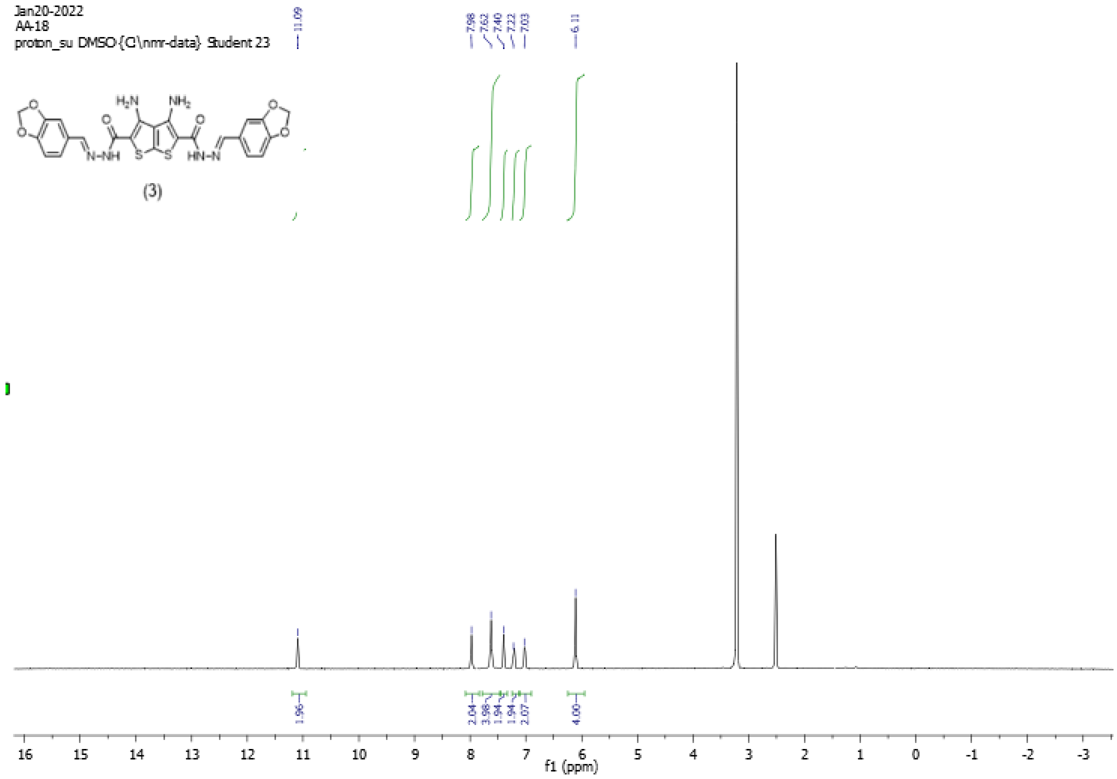

May31-2022
AA-18
c13_su DMSO{C:\nmr-data} Student 3

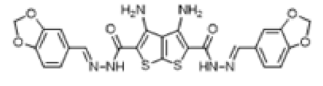

(3)

^{13}C NMR (101 MHz, DMSO) δ 165.29, 151.08, 149.16, 148.45, 142.63, 129.25, 127.37, 123.47, 109.09, 106.10, 101.96, 98.02.

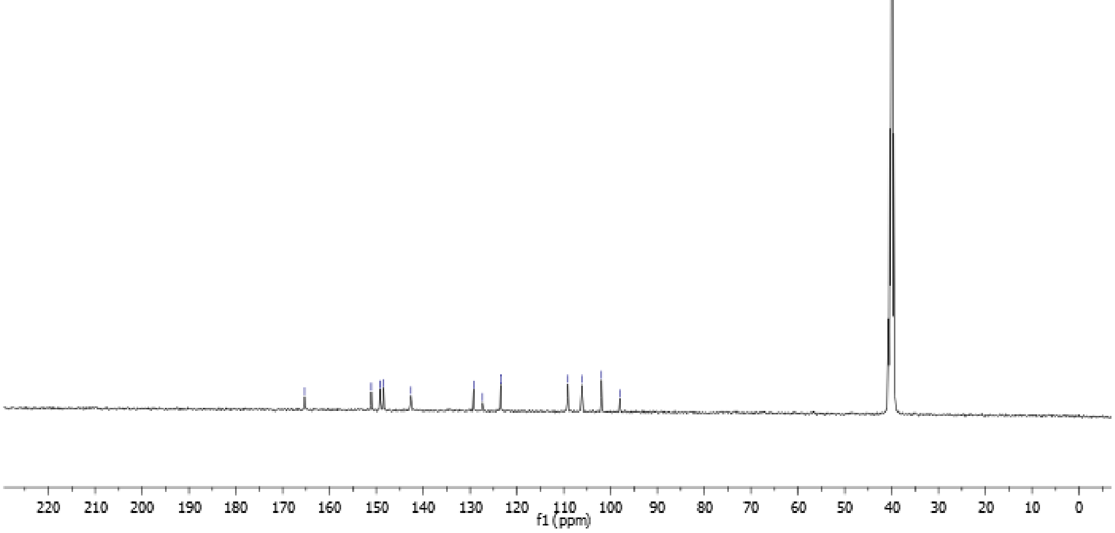

^{1}H-NMR and ^{13}C-NMR of compound **(4)**

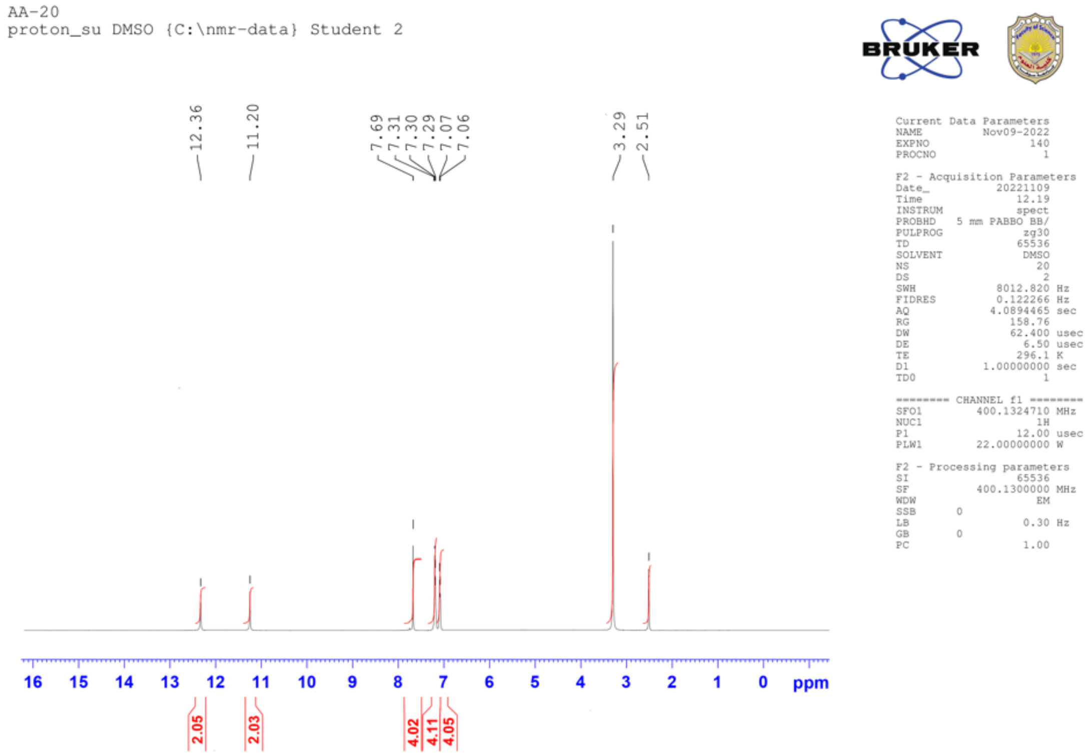

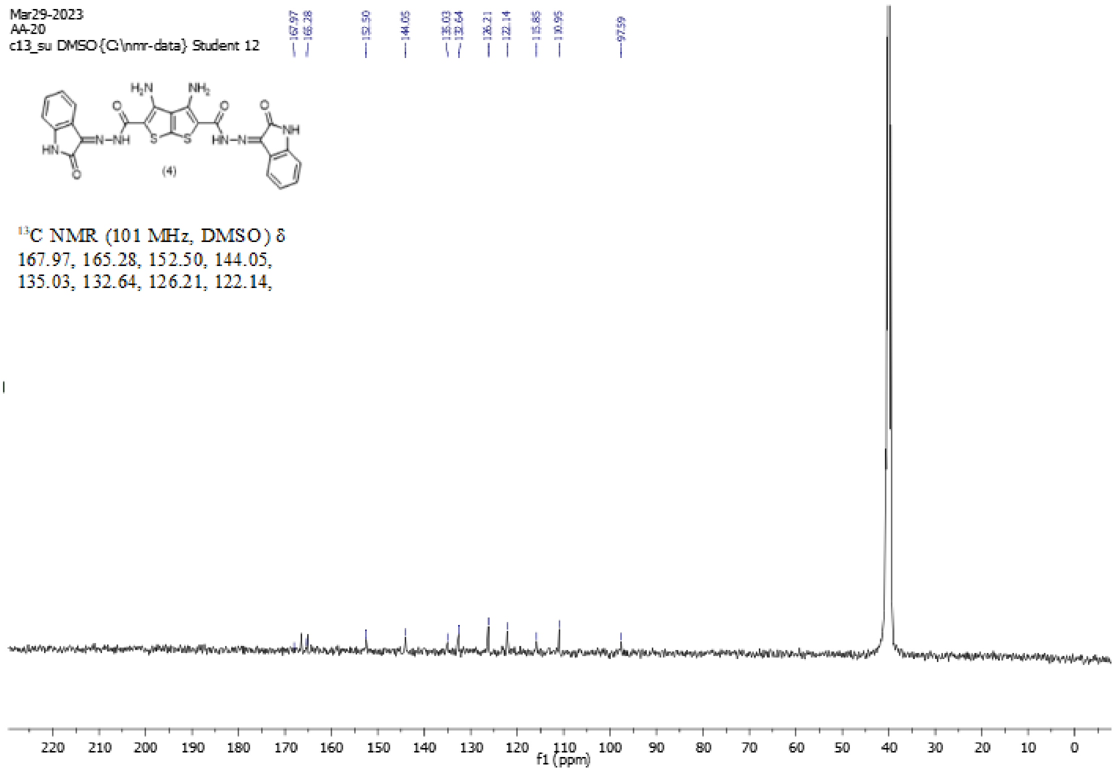

^{1}H-NMR and ^{13}C-NMR of compound **(5)**

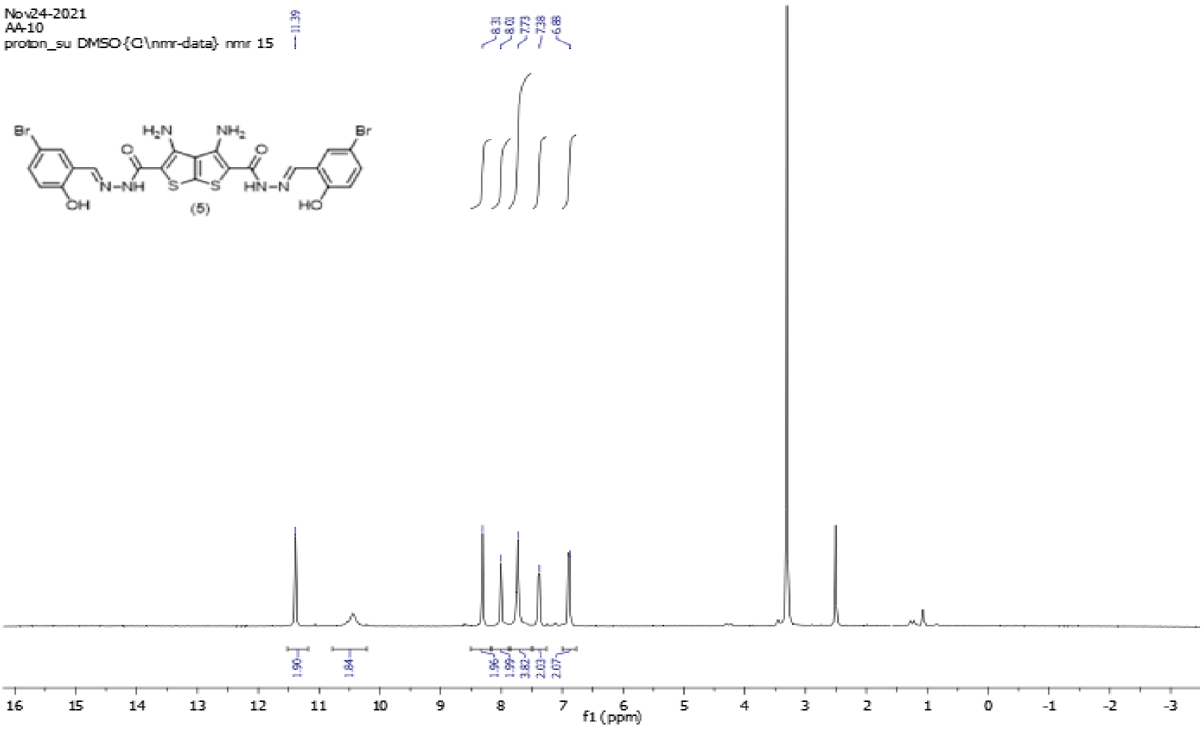

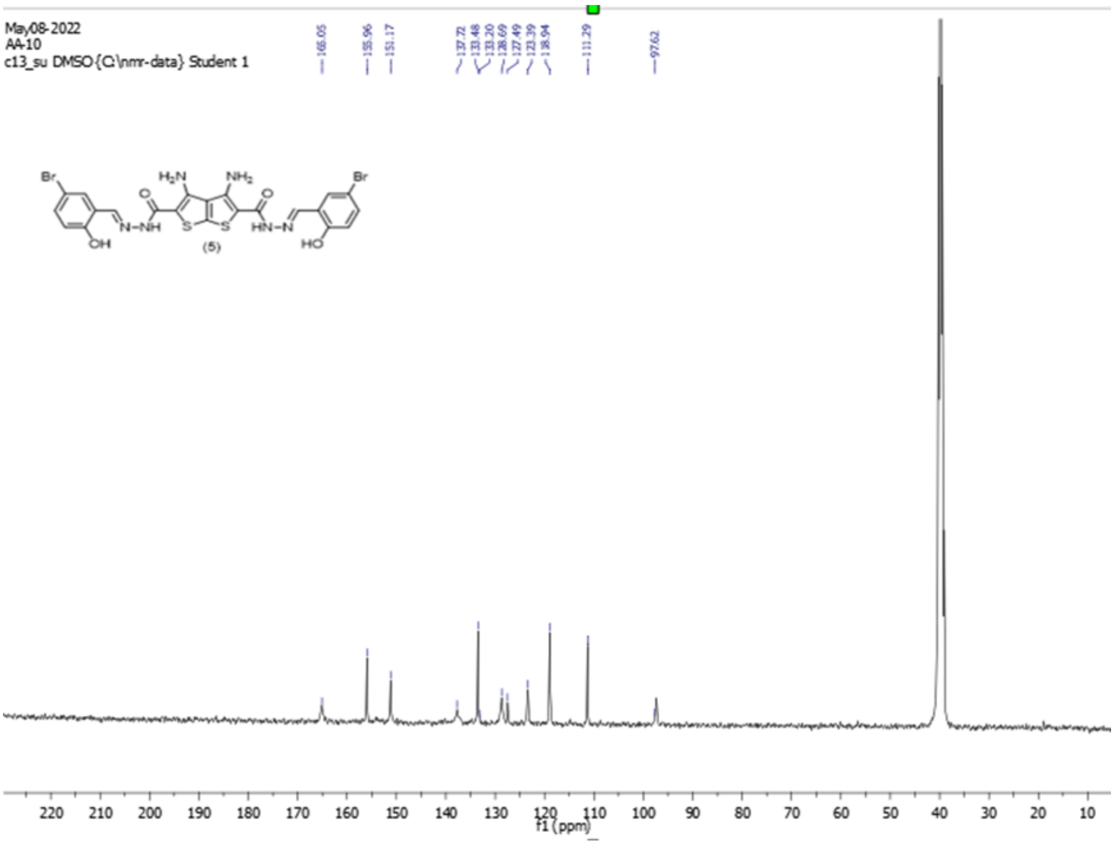

^{1}H-NMR and ^{13}C-NMR of compound **(6)**

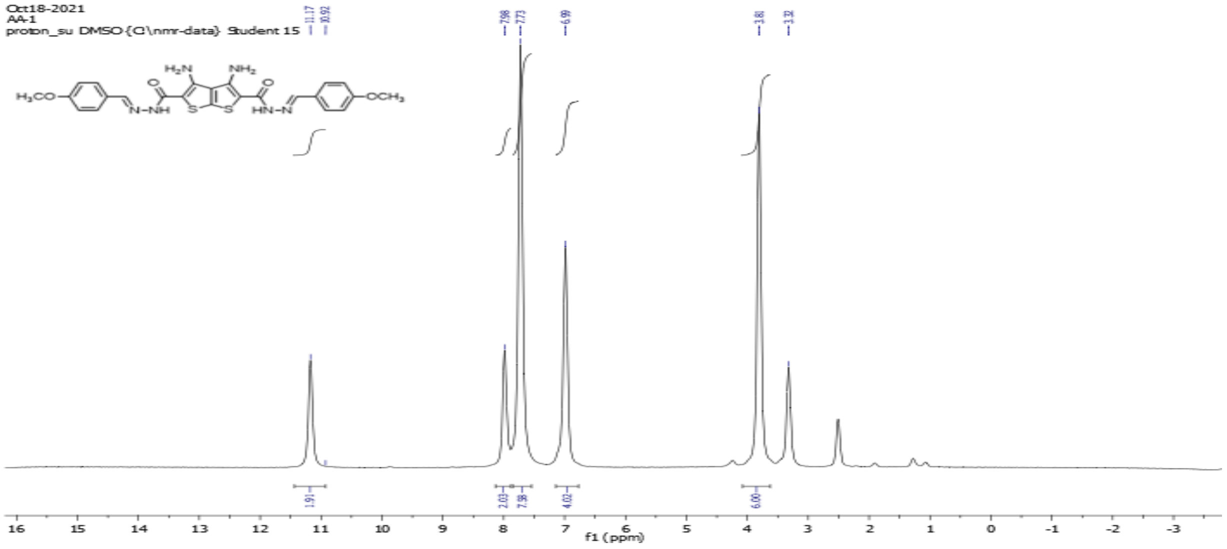

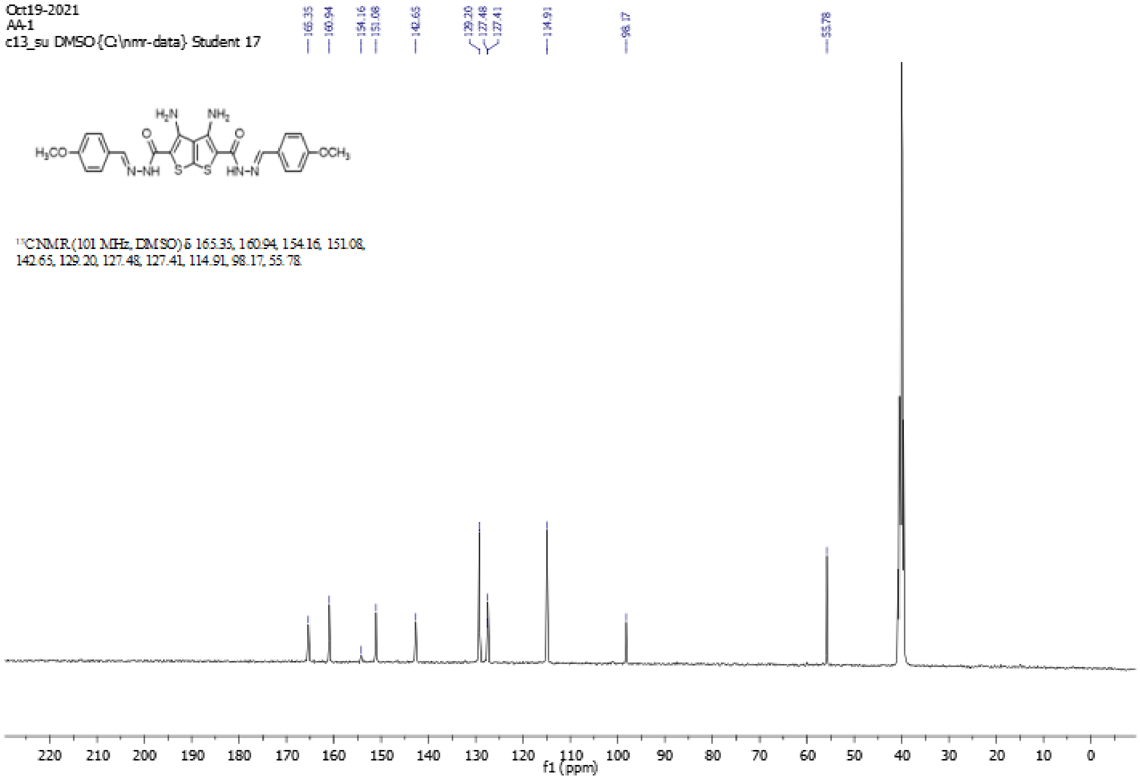

^{1}H-NMR and ^{13}C-NMR of compound **(7)**

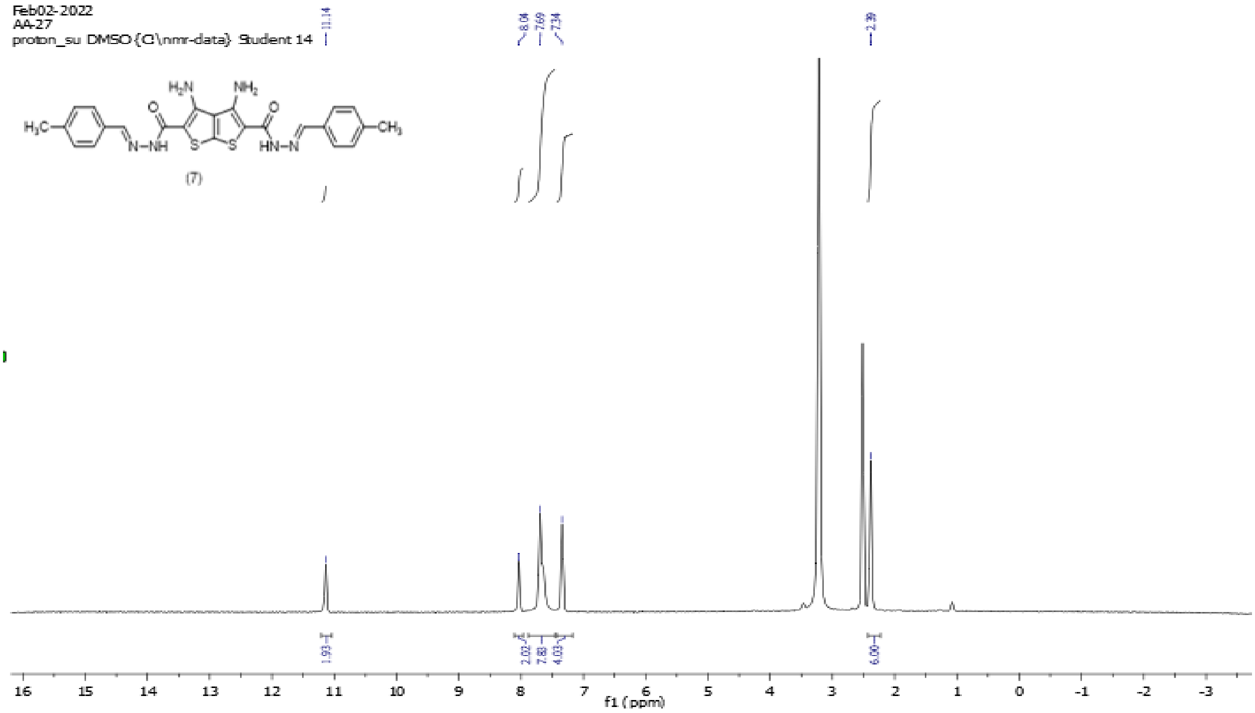

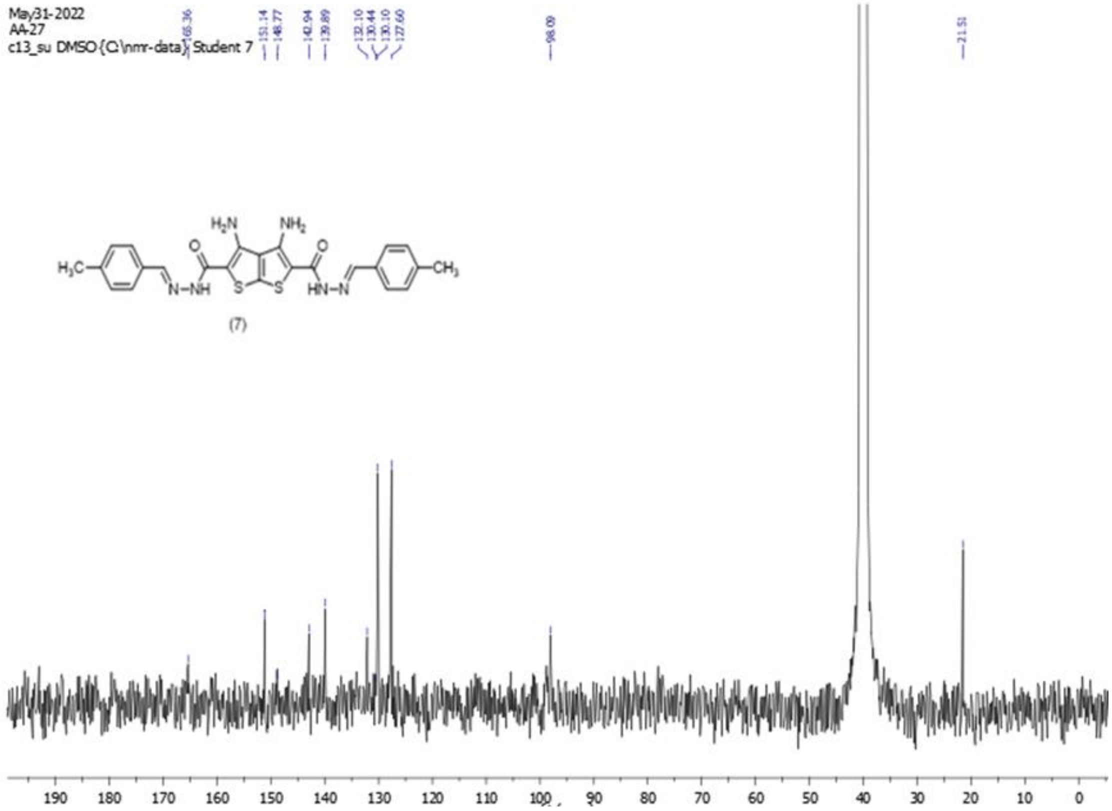

^{1}H-NMR and ^{13}C-NMR of compound **(8)**

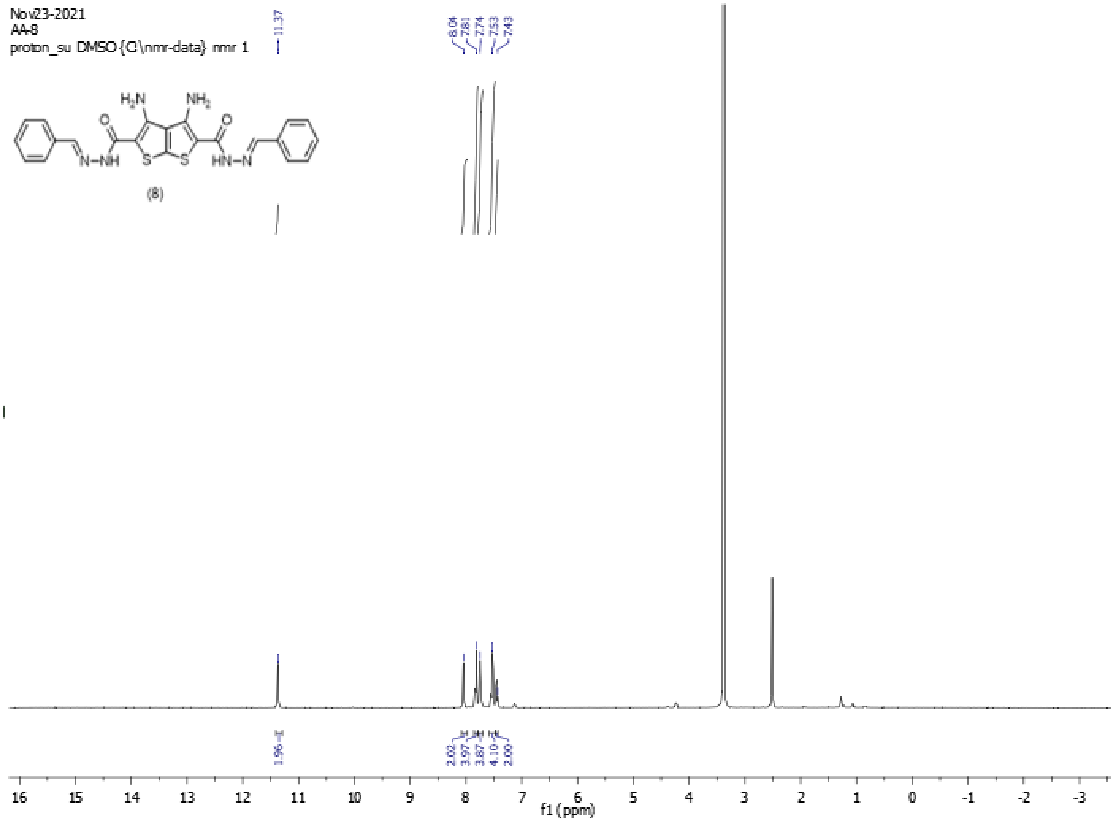

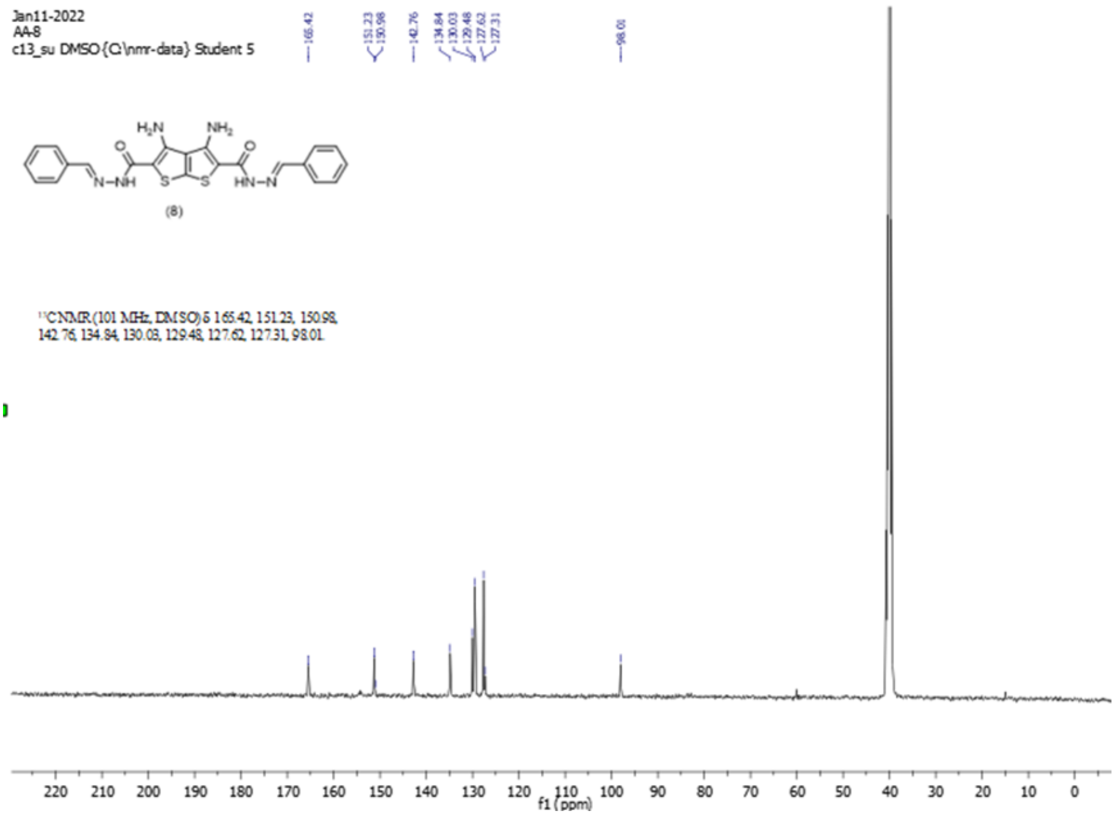

Supplement: Supplementary file 1 — Supplementary Information. [file 41598_2024_83994_MOESM1_ESM.pdf]
